# Expression of down-regulated ERV LTR elements associates with immune activation in human small-cell lung cancers

**DOI:** 10.1186/s13100-023-00290-w

**Published:** 2023-03-14

**Authors:** Marco Russo, Sara Morelli, Giovanni Capranico

**Affiliations:** Department of Pharmacy and Biotechnology, Alma Mater Studiorum, University of Bologna, via Selmi 3, 40126 Bologna, Italy

**Keywords:** Small-cell lung cancer, Transposable elements, Innate immune response, Cytoplasmic RNA sensors, Cancer treatment

## Abstract

Small-cell lung cancer (SCLC) is an aggressive cancer characterized by immunosuppressive features leading to poor responses to current immunotherapies. Activation of transposable elements (TE) can trigger an innate immune response, which can synergize with immunotherapeutic protocols in patients. However, TE activity in relation to immune gene response is not fully known in human SCLC. Here, we compared TE expression in 104 human SCLC and 24 normal tissues and established their involvement in innate immune responses. We observed that different intergenic TEs, mainly endogenous retroviral (ERV) families, are deregulated in SCLC. Similarly to other cancers, we detected a subset of LTRs that correlate with innate immune gene signatures and cytosolic RNA sensors, such as RIG-I. These LTRs are downregulated in SCLC tumors vs. normal tissues, and are mainly located at transcriptional repressed regions, marked with H3K4me2 in different cell lines. Analyses of different genomic datasets show that chromatin repression is likely due to de-methylase LSD1 activity. Moreover, high expression levels of ERV LTRs predict a better survival upon chemotherapy of SCLC patients. The findings reveal a specific pattern of TE-mediated activation of innate immune genes in SCLC, which can be exploited to establish more effective immunotherapeutic combinations.

**Supplementary Information:**

The online version contains supplementary material available at 10.1186/s13100-023-00290-w.

## Introduction

Small-cell lung cancer (SCLC) is an aggressive tumour that accounts for almost 15% of all lung tumours with a 5-7% of survival rate after 5-years from the diagnosis. Chemotherapy treatments are firstly effective, but then tumour recurrences are drug-resistant commonly leading to death within few months [1–3]. SCLC patients also show mostly discouraging responses to current immunotherapeutic treatments due to a high level of immunosuppression and low T-cell infiltration, even though the disease is characterized by a high mutational burden [4]*.* Recently, the association of immune checkpoint blockade (ICB) treatment with inhibitors targeting the DNA damage response (DDR) pathway has been shown to be effective in multiple murine SCLC models [5]. DDR inhibitors or DNA-interacting agents can increase the surface expression of PD-L1 and/or activate the cGAS-STING pathway, mediated by cytosolic DNA sensors, leading to Type I interferon stimulated gene (ISG) expression [6, 7] and tumour-infiltrating cytotoxic T-lymphocytes [5, 8]. Therefore, triggering the cellular innate immune response can act synergistically with immunotherapeutic approaches to achieve longer survival of SCLC patients. However, cGAS and STING expression is significantly reduced in human SCLC as compared to normal lung and other lung cancers leading to a marked impairment of the cGAS/STING pathway [9]**.** These results highlight the need to develop new strategies to stimulate innate immune genes in unresponsive SCLC.

Pharmacological approaches aimed at activating an anti-viral response in tumours, based on the upregulation of transposable elements (TE) as “viral mimicry”, has recently become an active research topic with promising results [10–12]. TEs are ubiquitous, long-standing genetic elements of eukaryotic genomes, capable of mobilization throughout the entire cell genome by an autonomous replication mode. They are commonly divided into two main classes: Class I is comprehensive of endogenous retroelements, namely long terminal repeats (LTRs), long interspersed elements (LINEs) and short interspersed elements (SINEs), which replicate through an RNA intermediate and a “*copy and paste*” mechanism. Class II includes DNA transposons, which replicate through a DNA intermediate and a “*cut and paste*” mechanism [13]. In several cases, TEs overlap with expressed genes in the human genome, therefore causing difficulties in distinguishing true TE-derived reads from expressed gene-derived reads in transcriptome sequencing approaches [14].

Over evolutionary time, due to their intrinsic genetic properties and activities, certain TEs went through exaptation in *cis*-regulatory elements and co-opted for the tuning of essential gene functions [13]. Interestingly, a mammalian lineage-specific subset of endogenous retrovirus (ERVs) turned out to act as interferon-inducible enhancers essential for the activation of the AIM2 inflammasome [15]. However, the majority of TEs have undergone silencing by the host genomes in order to restrict their pervasive and potentially deleterious de novo insertions as well as transcriptional and post-transcriptional effects. RNA-Seq data analyses of genome-wide quantification of TE expression using The Cancer Genome Atlas (TCGA) database showed overexpression of specific TE subfamilies in tumour vs matched normal samples and an association with antiviral and DNA damage responses [14]. Moreover, treatment of glioblastoma cells with demethylating agents resulted in increased TE expression and antigenicity through the presentation of novel TE-derived peptides on class I MHC [14]. Analogously, endogenous retroelement overexpression in ovarian cancer cell lines treated with DNA methyltransferase inhibitors (DNMTis) could trigger an antiviral response mediated by cytosolic dsRNA sensing [10]. Chemotherapy-induced TE expression has been demonstrated to activate the MDA5 pathway leading to an inflammatory response required for hematopoietic regeneration [16]. These findings altogether are consistent with a mechanism of induction of an innate immune response mediated by the sensing of TE-derived nuclei acids in the cytosol of cancer cells due to epigenetic dysregulation of genomic TE copies [17, 18]. However, little is known of TE expression changes and induction of immune response in human SCLCs.

Thus, we here analysed TE families in 104 human SCLC samples and 24 normal lung tissues, along with patterns of immune gene expression levels, restricting the study to intergenic TEs to avoid ambiguity in assigning sequence reads to overlapping TEs and gene transcripts. We found that expression levels of certain intergenic TEs, which are downregulated in SCLC tumors with respect to normal tissues, correlate with innate immune gene signatures. Moreover, high expression levels of these TEs predict a better survival upon chemotherapy of SCLC patients. The findings reveal a specific scheme of TE-mediated activation of innate immune genes in SCLC, which can be exploited to establish more effective immunotherapeutic combinations.

## Results

### Intergenic TE expression is deregulated in human SCLCs

**Fig. 1:**
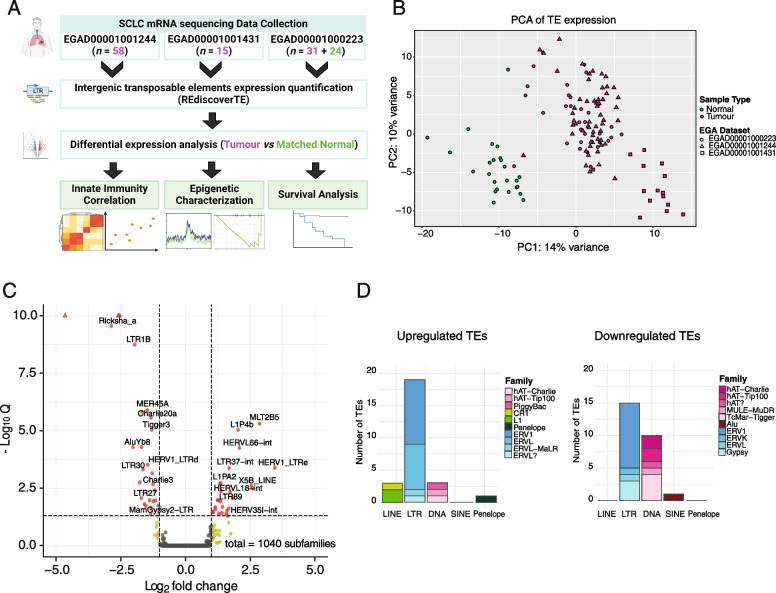
TEs are deregulated in human SCLCs. **A** Design of data analyses performed starting from collecting RNA-seq data from three public datasets of SCLC samples. Intergenic transposable element expression was quantified with *RediscoverTE* [14] and differential expression analysis was performed. Then we characterized deregulated TEs involvement in innate immune induction, epigenetic changes in SCLC and patient survival. Created with BioRender.com. **B** PCA plot of SCLC sample datasets based on transposable element expression. Samples are 104 SCLC from three different EGA Datasets (magenta circles, triangles and squares) and 24 normal lung (green circles) samples. **C** Volcano plot of differentially expressed intergenic TEs in 24 SCLC vs matched normal samples. X-axis: log_2_ fold change values of differential expression. Y axis: -Log_10_(q-value). Points correspond to intergenic TE copies included in DNA, LTR, LINE, SINE, Penelope and retroposons classes (*n* = 1040). Labels are assigned only to intergenic differentially expressed TEs (*n* = 52). Red points correspond to differentially expressed TEs with log_2_ fold change ≥ + 1 or log_2_ fold change ≤ − 1 and *q*-value ≤0.05. Yellow points correspond to differentially expressed TEs with log_2_ fold change ≥ + 1 or log_2_ fold change ≤ − 1 and q-value ≥0.05**. D** Stacked bar plot of the number of upregulated (left) and downregulated (right) transposable element copies summarized for each family and grouped for each class in SCLC tumour vs matched normal samples. Gradient colour is shown according to the legend

We characterized the landscape of TE expression in SCLCs by using RNA-Seq data from a cohort of tumours and matched normal tissues [19], and two cohorts of tumour samples [1, 20] for a total of 104 cancer and 24 normal samples. Quantification of TE transcripts was performed using the *REdiscoverTE* tool [14], which aggregates the expression of multiple TE copies under the subfamily to which they belong. The tool also gathers TE copies into intergenic, exonic or intronic types based on the host genomic locus. Thus, since we observed that the overall expression of the main TE classes basically stems from intergenic regions (Fig. S1A) and to further exclude ambiguous annotation of reads originating from host gene transcripts or chimeric transcripts, we have restricted the analyses to intergenic TEs only (Fig. 1A). A Principal Component Analysis (PCA) of intergenic TE expression reveals that normal lung and SCLC tissues clustered into two well-separated areas based on overall TE expression (Fig. 1B), in agreement with the same analysis of gene expression (Fig. S1B). Therefore, intergenic TE transcriptome is overall altered in SCLC with respect to normal lung.

Next, we investigated how TEs are differentially expressed in tumour samples as compared to matched normal tissues. Firstly, raw counts of repeated sequences were used to filter out subfamilies with few counts. Then, a paired differential expression analysis was performed to account for patient-specific differences (Fig. S1C). The analysis results in 1040 intergenic TE families belonging to six classes: DNA transposons, long-terminal repeats (LTRs), long-interspersed repeats (LINEs), Penelope elements, short-interspersed repeats (SINEs) and retroposons (SVAs). Of them, 52 (5%) TE families were deregulated in SCLC vs. normal lung (Table S1), equally divided into upregulated and downregulated (Fig. 1C). In addition, we noticed patient-specific TE expression levels, with a subgroup of patients characterized by an overall higher expression of many deregulated TEs (Fig. S1C). However, TE expression levels differ between tumour and normal samples with a high reproducibility (Fig. S1D). Notably, deregulated TEs belong to all the above-mentioned TE classes, apart from SVA elements (retroposons). The majority of either upregulated and downregulated intergenic TEs belongs to LTRs (*n* = 19 and 15, respectively) or DNA transposons (*n* = 3 and 10, respectively) (Fig. 1D). SINE and LINE and Penelope elements are less frequent among the deregulated TEs. Interestingly, most of deregulated LTRs belong to the endogenous retroviral ERV1 family (Fig. 1D). Since TE expression is prevalent at intergenic regions (Fig. S1A), we observed that performing the differential analysis using global level of TE expression leads to similar results in terms of deregulated candidates (Table S2).

### Expression of downregulated ERVs correlates with immune response gene pathway

**Fig. 2:**
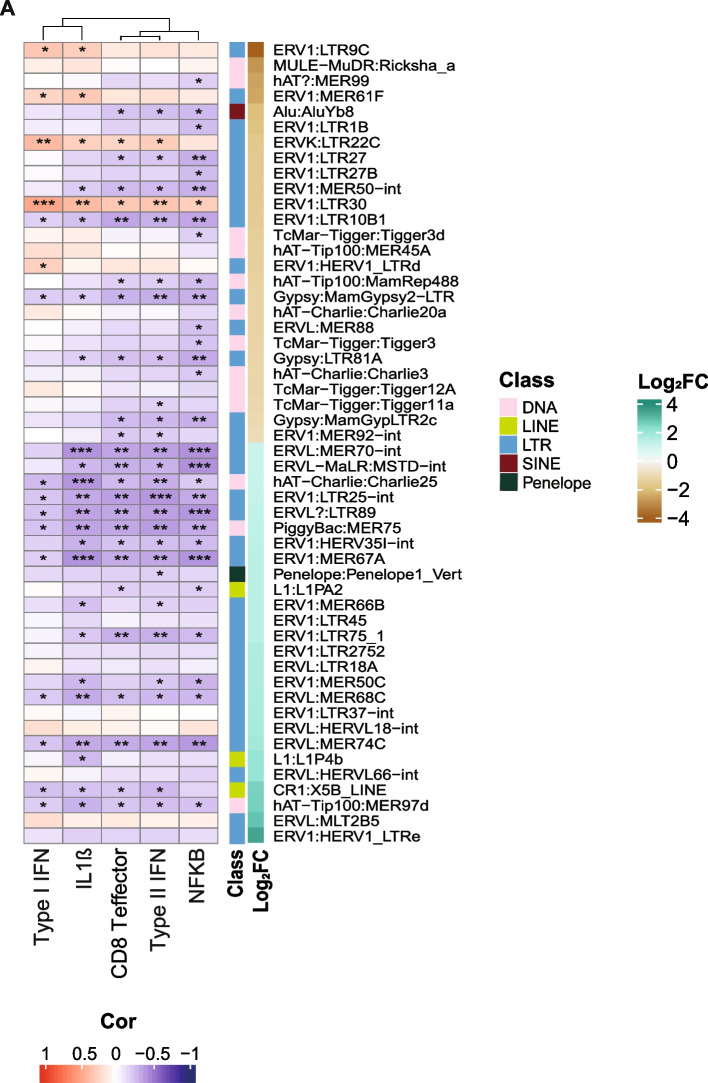
Downregulated TEs correlates with innate immune activation. **A** Heatmap of correlation between expression of differentially-expressed TEs (rows) and ssGSEA scores of immune response genes (columns). Heatmap annotation: Spearman’s *rho* correlation coefficient (Cor). Negative to positive correlation values correspond from blue to red, respectively. *p*-values: * 0.001-0.05, ** 0.00001-0.001, *** < 0.00001. None, > 0.001. Log_2_FC annotation: log_2_ fold change values of differentially expressed TEs, ranked from the negative (yellow) to the positive (green) values. Class annotation: TE class colour as shown

We then wondered whether the expression levels of deregulated TEs were associated with activation of immune genes in SCLC. Firstly, making use of immune response-related genes and the MSigDB Hallmark collection, we computed ssGSEA enrichment scores for each of the 104 tumour samples. Then, we determined a correlation factor between the enrichment score and deregulated TE expression. We found that expression levels of TE subfamilies upregulated in tumour samples compared to matched normal ones were anti-correlated with immune response signatures (Fig. 2A, Fig. S2A). Conversely, among downregulated intergenic TEs, the expression levels of ERV subfamilies LTR30, LTR22C, LTR9C, MER61F, the intergenic expression of which is prevalent comparing to intragenic one (Fig. S3A), and, to a lower extent, HERV1_LTRd, were positively correlated with immune response gene signatures, particularly for Type I Interferon pathway (Fig. 2A). LTR30 is positively correlated with all the immune-related gene sets (Fig. 2A). Consistently, MSigDB Cancer Hallmark analyses showed a strong correlation with interferon responses and inflammatory signatures for the above TEs only, whereas many upregulated TEs showed an anticorrelation with the signatures (Fig. S2A). A few of upregulated TEs (such as HERVL18_int) were slightly associated with immune features, but not at significant levels (Fig. 2A and S2A). Then, we asked whether the observed downregulation of ERVs involves all the elements of the same subfamily or only some of them. Therefore, we firstly determined TE expression levels for each genomic copy using TElocal tool [21]. Then, we performed a differential expression analysis for each copy of TE subfamily. The results showed that LTR30, LTR22C and LTR9C subfamilies are characterized by many significantly downregulated copies and only a few upregulated, while MER61F and HERV1_LTRd showed only downregulated copies (Fig. S4A-E). An upregulated ERV, MER74C, is instead characterized by mostly upregulated loci (Fig. S4F). Altogether, results from the differential expression analysis at locus level identified specific copies potentially accounting for the overall deregulation observed at subfamily level (Table S3). Correlation of the expression at these copies of downregulated ERV subfamilies and ssGSEA enrichment score of immune-related signatures resulted in a positive association for a few downregulated copies (Fig. S5A). Specifically, LTR30 (dup101) is the locus with the highest correlation scores, but also two copies of LTR9C (dup9 and dup137), MER61F (dup28 and dup93) and HERV1_LTRd (dup4 and dup5) and one copy of LTR22C (dup1) showed significant positive correlation scores.

### Cytosolic RNA sensor expression correlates with innate immune response genes

As the production of type I Interferon induced by TE expression can depend on cytosolic nucleic acid sensors [10, 16], we evaluated the association of 8 sensors with immune-related gene sets in SCLCs. We focused on well-recognized sensors of cytosolic dsRNAs such as melanoma differentiation-associated protein 5 (MDA5, also known as IFIH1) and retinoic acid-inducible gene I protein (RIG-I, also known as DDX58), which activate the mitochondrial antiviral-signaling protein (MAVS) and, ultimately, lead to the production of type I interferon [22]. In addition to the RIG-I/MDA5-MAVS pathway, we considered the endosomal Toll-like receptors specific for the recognition of ssRNAs and dsRNAs, TLR7/TLR8 and TLR3, respectively [17], and IFI16, which is a sensor of both RNA and DNA in cytosol [23–25]. We further considered the DNA sensors AIM2, which, after binding cytosolic DNA, mediate the production of IL-1β via inflammasome activation and the cGMP–AMP synthase (cGAS) sensor, which recognizes cytosolic DNA and activates STING (stimulator of IFN genes) [26] We found that expression levels of RNA cytosolic and endosomal sensors MDA5, RIG-I, TLR3 and TLR8, but also IFI16 are strongly correlated with the “Response to type I interferon” gene set, while cytosolic DNA sensors seem to be slightly associated with an innate immune response activation in SCLC tumours (Fig. S6A-I).

### ERV1 LTR30 and RIG-I are together a better predictor of immune response in SCLCs

**Fig. 3:**
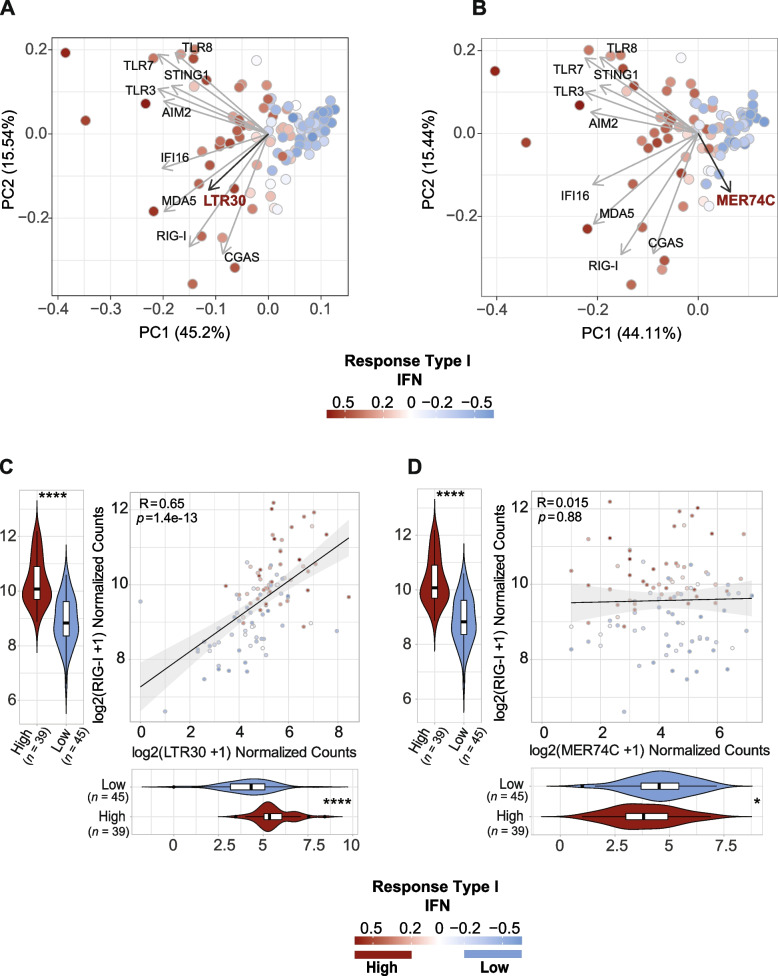
LTR30 expression strongly correlates with innate immune genes and RIG-I sensor expression. PCA plot of SCLC samples dataset based on innate sensors gene and **A)** LTR30 or **B)** MER74C expression. The PCA loadings describing the contribution to the components of each gene and TE are represented as labelled arrows. Points correspond to SCLC tumour samples (*n* = 104). Colour: Response to type I Interferon ssGSEA scores, as in legend. **C** Main: Scatter plot of correlation between log_2_ normalized counts of LTR30 expression (x-axis) and log_2_ normalized counts of RIG-I expression (y-axis). Points correspond to SCLC tumour samples (*n* = 104). Colour: Response to type I Interferon ssGSEA scores, as in legend. Spearman’s *rho* (R) and *p*-values of correlation (p) are reported. Down: Violin plot of LTR30 expression in samples with low levels of Response to type I Interferon (blue, NES < − 0.2) or with high levels of Response to type I Interferon (red, NES > 0.2). Left: Violin plot of RIG-I expression in samples with low levels of Response to type I Interferon (blue, NES < − 0.2) or high levels of Response to type I Interferon (red, NES > 0.2). Wilcoxon test *p*-values are reported: * 0.01-0.05, ** 0.0001-0.01, *** 0,00001-0.0001, **** < 0,000001. **D** Same as in **C)** but for MER74C

As certain LTR families and cytosolic RNA sensors are strongly associated to immune response genes, we then investigated whether the combination of expression of intergenic LTR30, LTR9C, LTR22C and MER61F families, and cytosolic RNA sensors is a better predictor of type I interferon response pathway in SCLCs. PCA analyses of cytosolic sensors and TE expression showed that higher expression of LTR30 or LTR9C, LTR22C and MER61F, on one side, and cytosolic RNA sensors, on the other, predicted a higher response to type I interferons (Fig. 3A, Fig. S7A). We did not detect such a correlation with TEs that are upregulated in SCLCs as compared with matched normal samples, such as MER74C (Fig. 3B).

The best correlation scores were observed with LTR30 element and RIG-I sensor (Fig. 3A, Fig. S7A). We clearly observed that LTR30 expression levels best fitted with RIG-I expression (Spearman’s *rho* = 0.65) and also that higher expression levels of both characterize tumour samples with positive enrichment (NES > 0.2) of Type I interferon response gene set. Consistently, tumour samples characterized by negative enrichment (NES < − 0.2) show lower expression of both LTR30 and RIG-I (Fig. 3C). The same trend also characterized the correlation with TLR sensors, but also with STING (Spearman’s *rho* = 0.48) and IFI16 (Spearman’s *rho* = 0.56) (Fig. S8A-D). SCLC samples with positive enrichment of type I interferon response also showed a higher expression, but to a lower extent, of LTR9C, LTR22C and MER61F subfamilies and cytosolic RNA sensors. On the contrary, the expression of TE subfamilies found to be upregulated in tumours vs normal tissues, such as MER74C (Fig. 3D), show no correlation with response to type I interferon. Interestingly, a higher expression of MER74C was associated to a minimal response to type I interferon. Therefore, the expression of either LTR30 and other TE subfamilies, and RIG-I and other RNA sensors is associated to an innate immune response in human SCLCs.

### Demethylation of H3K4me2 likely drives repression of intergenic ERVs in human SCLCs

As intergenic LTR30, LTR9C, LTR22C and MER61F are downregulated in SCLC tumors with respect to normal samples, and their expression correlated with innate immune response, we next investigated the mechanism of LTR transcriptional repression in SCLCs by further bioinformatic analyses of biological datasets. In particular, we determined prevalent histone modifications and Transcription Factor Binding Sites (TFBS) at all deregulated intergenic LTRs by performing a locus enrichment analysis with the LOLA tool [27] of the epigenome Cistrome database [28]. As Cistrome datasets constitute a collection of ChIP-seq data from different cancer cells (but not SCLC cells), the statistical significance of the analyses does not rely only on *p*-values and percentages of support of individual marks (Fig. S9A, as described in caption), but it is based on the recurrent frequency of enriched marks across several cell lines.

**Fig. 4:**
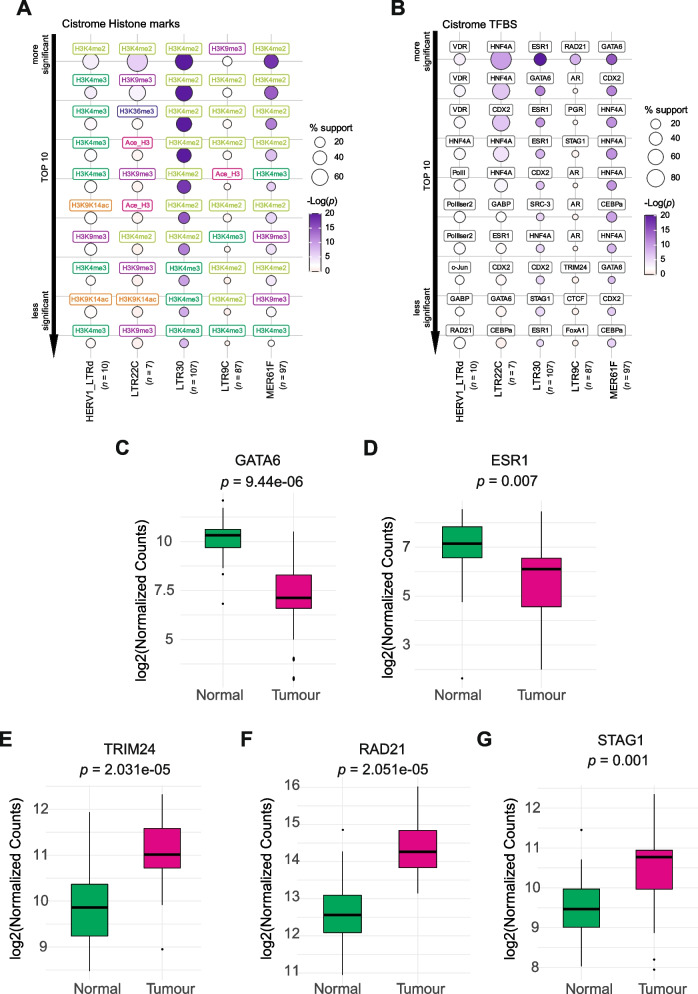
Downregulated TEs are associated with specific histone marks and transcriptional-suppressed loci. Bubble plot of top ten ranked **A)** Cistrome Epigenome marks and **B)** Cistrome TFBSs associated to deregulated LTR elements. Bubble colour: -Log(*p*-value), as in legend. Bubble size: “support” percentage, expressed as the ratio between the number of intergenic differentially-expressed LTRs loci overlapping with Cistrome marker loci and the number of all intergenic LTR loci. **C-G)** Boxplots of GATA6 (**C**), ESR1 (**D**), TRIM24 (**E**), RAD21 (**F**) and STAG1(**G**) gene expression in 24 tumour and matched normal samples expressed as log_2_(normalized counts expression + 1). Paired t-test *p*-values are reported

Firstly, we observed that intergenic LTR30 elements (and to a lower extent MER61F and LTR22C) co-localize with di-methylation and tri-methylation of histone 3 at Lysine 4 (H3K4me2 and H3K4me3, respectively) (Fig. 4A), which are signals of transcriptionally active chromatin. The results suggested that repression of intergenic LTR30s in SCLC may be due to alterations of H3K4 methylation levels. Many analysed LTRs showed no significant enrichment, except for LTR10B1 and MER50-int subfamilies that are associated to the repressive tri-methylation of histone 3 at Lysine 9 (H3K9me3) mark (Fig. S10A). Conversely, loci associated to upregulated LTRs in SCLC are overall enriched in repressive histone markers (Fig. S10B), indicating that epigenetic changes may involve those histone marks leading to upregulation in SCLCs.

As H3K4me2 and H3K4me3-enriched regions more often overlap with transcription factor binding regions [29], we next performed enrichment analyses with Cistrome TFBS database to determine the specific factors that bind to intergenic LTRs deregulated in SCLCs. While many analysed LTRs showed no significant enrichment (Fig. S11A), we observed a significant association between downregulated LTR30 and ESR1 (Estrogen Receptor 1) binding sites, and, to a lower extent, GATA6 (GATA binding protein 6) sites (Fig. 4B). In addition, we observed significant colocalization for LTR22C (HNF4A, Hepatocyte Nuclear Factor 4 Alpha) and MER61F (GATA6 and HNF4A). Among the upregulated TEs, we observed significant associations for HERVL18-int (STAG1, RAD21), LTR2752 (RXR), MER66B (TRIM24) (Fig. S11B). Notably, we observed that GATA6 and ESR1 genes were significantly downregulated in SCLCs as compared with normal samples (Fig. 4C-D), while STAG1, TRIM24 and RAD21 showed an upregulation (Fig. 4E-G) suggesting that expression alterations of these transcription factors may affect the expression of the studied intergenic ERV LTRs. We performed a transcription factor binding motif discovery for intergenic loci of LTR30, LTR22C and MER61F to further validate the potential association with these transcription factors. Interestingly, we detected a very significant presence of ESR and GATA binding motifs (Fig. S12, p-val 10e-197 and 10e-182 respectively) but the most enriched motifs regarded immune-related IRF and STAT transcription factors (IRF8, p-val 10e-248, IRF4, p-val 10e-245 and STAT5a p-val 10e-243). We obtained less significant but similar results for MER61F loci (Fig. S13), while for LTR22C we obtained unreliable potential motifs due too low *p*-values (Fig. S14).

**Fig. 5:**
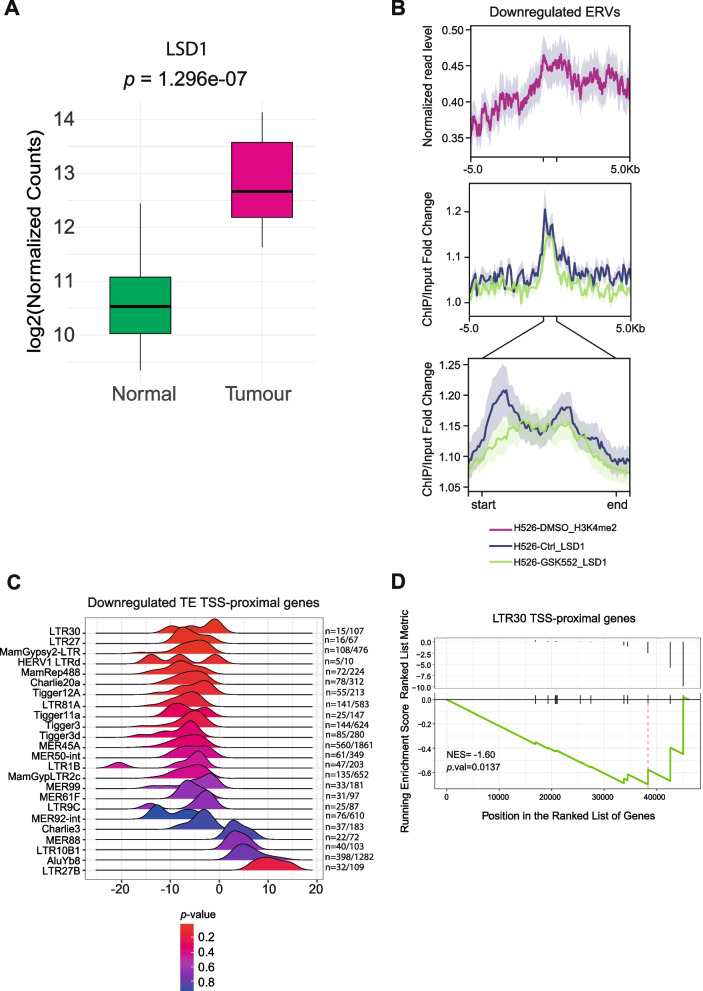
Downregulated TEs are associated with LSD1 occupancy and transcriptional repression in SCLC. **A** Boxplot of LSD1 gene expression in 24 tumour and matched normal samples expressed as log_2_(normalized counts expression + 1). Paired t-test *p*-values are reported. **B** Mean of Normalized read levels for H3K4me2 over downregulated ERV genomic loci (purple) in H526 SCLC cell line and mean genomic signal of ChIP/input ratio over same loci for LSD1 in H526 SCLC cell line in untreated (blue) and treated with LSD1 inhibitor (green) conditions. **C** Ridgeline plot showing the distribution of relative ranking metric values expressed as Wald test statistic values from GSEA (x-axis) of genes proximal to downregulated TEs (y-axis) in SCLCs. Colour: GSEA *p*-values, as in legend. For each TE, the ratio between the number of loci located upstream control regions of genes over the total number of loci is reported. **D** GSEA plot of genes, in the upstream regulatory regions of which LTR30 elements are localized (*n* = 4). Top: position of genes along the ranked list of genes with relative ranking metric values expressed as Wald test statistic values. Bottom: running enrichment score line plot; vertical red line indicates the peak of the plot at which the ES is computed. Normalized enrichment score (NES) and adjusted *p*-values are indicated

As intergenic LTR30 elements and other ERVs are commonly associated with H3K4me2 mark, we wondered whether de-regulation of this histone modification may contribute to TE repression in SCLCs. Histone de-methylase LSD1, responsible for methyl group removal from H3K4 [30], was shown to play a role in histone methylation patterns in SCLC and to be a promising drug target [31]. Thus, we investigated its expression levels in SCLC and found that LSD1 is overexpressed in tumour samples as compared to normal lung (Fig. 5A). In addition, using H3K4me2 and LSD1 ChIP-seq data from the SCLC H526 cell line [31], we observed a significant association of intergenic loci of downregulated ERV with H3K4me2 histone modification and LSD1, and that treatment with an LSD1 inhibitor reduced its binding to these loci (Fig. 5B).

We performed the same analysis considering downregulated ERV loci that were found expressed and downregulated with locus specific TE quantification, and we observed that some loci are associated more than others to H3K4me2 histone modification and that likely the same loci seem to be the most associated to LSD1 histone de-methylase (Fig. S15A, B). As the results indicate that LSD1 can play a role in the repression of intergenic ERV LTRs, we then wondered whether the expression of LTR-proximal genes was also downregulated. Then, we collected the genes in the upstream regions (within 20 kbp from TSS) of which, the repressed LTR elements are located, and performed a Gene Set Enrichment Analysis for each repressed LTR subfamily. We found that many downregulated LTRs are often located upstream (control) regions of genes that are under-expressed in SCLCs as compared with normal lung tissues (Fig. 5C). Again, the most enriched intergenic TE is ERV1 LTR30 subfamily (Fig. 5D). The results indicate that downregulated intergenic ERV LTR elements are likely embedded in repressed chromatin including TE elements and proximal genes. In addition, since SCLC is characterized by hypermethylation at many promoter sites [32], we investigated DNA methylation status of intergenic LTRs in SCLC and normal lung in order to find any correlation between DNA methylation and LTR30 expression. However, we did not find any evidence of methylation changes in LTR30 genomic regions in SCLCs (Fig. S16 A-C) that may suggest an epigenetic regulation that is observed for protein coding genes (e.g. STING, Fig. S16D).

Altogether, the findings indicate that repression of ERV LTR subfamilies in SCLC are likely due to epigenetic repression of chromatin in SCLC tumours. Downregulated LTR30, LTR22C, LTR9C and MER61F are likely associated with reduced H3K4me2 levels due to LSD1 overexpression in SCLC vs normal lung.

### High ERV1-LTR30 expression predicts a higher efficacy of chemotherapy in SCLC patients

**Fig. 6:**
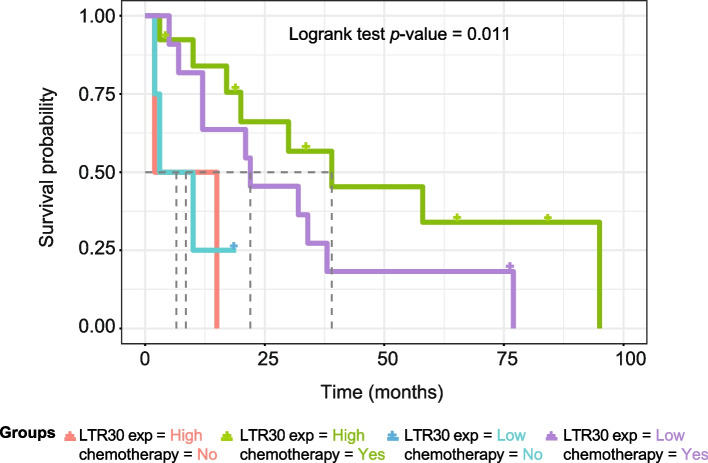
LTR30 expression predicts a higher efficacy of chemotherapy in SCLC patients. Kaplan-Meier plot of survival estimates of SCLC patient subgroups (total *n* = 30) depending on expression levels of LTR30 and chemotherapy. Log rank test *p*-values are reported. Colour codes are

Since innate immune genes activation in SCLC has been proposed to act synergistically with immunotherapy approaches to promote longer survival in patients [5], we wondered if high expression levels of LTR30, LTR22C, LTR9C and MER61F, that we observed correlate with innate immune response signatures in SCLC, can predict a favourable prognosis in SCLC patients treated with chemotherapy as well. Therefore, we performed a survival analysis of the SCLC patients that had not been treated with drugs before surgical tumour resections and grouping them based on LTR expression levels and on chemotherapy after the resection (total *n* = 30). The results clearly showed that patients with high expression levels of ERV1 LTR30 show a longer survival rate in case they underwent chemotherapeutic treatments, but not if they were not treated with drugs (Fig. 6). Patients with low ERV1 LTR expression show a shorter survival rate regardless of chemotherapeutic treatment (Fig. 6). Similar results were obtained for LTR9C, whereas LTR22C and MER61F effects were not significant (Fig. S17A-C). Therefore, the analyses underline the clinical impact of ERV LTR30 and LTR9C expression levels on SCLC response to chemotherapy.

## Discussion

Our findings provide unexpected insights into the emerging implications of LTR expression on the modulation of innate immune responses in human SCLC. To overcome the challenge of a precise detection and measurement of the levels of short-read sequences from repetitive regions overlapping transcribed genes, we have here used the recently developed method *REdiscoverTE* [14] to quantify the expression of all repetitive sequences, including TEs, focusing on intergenic loci only. Moreover, we have demonstrated the reliability of the technical approach comparing it with a robust locus-level quantification method [21, 33]. We used RNA-seq data from 104 human SCLC and 24 normal lung tissues [1, 19, 20]. Differential expression analysis highlighted a subset of intergenic deregulated TE subfamilies, equally distributed into upregulated and downregulated in SCLC vs normal lung, which are mainly LTR elements belonging to distinct ERV families (Fig. 1). Surprisingly, we found that expression levels of specific downregulated (with respect to normal tissues) ERV1s, namely LTR30, LTR9C, LTR22C and MER61F subfamilies, are significantly correlated to the activation of innate immune response in SCLCs. Moreover, we found that specific cytosolic and endosomal RNA sensors, such as RIG-I (Figs. 2 and 3), are also correlated with immune gene response, suggesting a potential combined role in inducing an innate immune response in SCLCs [34]. On the contrary, expression of TE subfamilies upregulated in tumours vs normal lung [14] did not correlate with immune responses in SCLC. LTR30 and RIG-I expression levels are together a better predictor of innate immune responses, and high LTR30 expression levels positively impact on patient survival upon chemotherapy (Fig. 6). Locus-level quantification of TE expression confirmed our findings about TE deregulation in tumours and some TE correlation with innate immune signatures, showing that it is likely restricted to specific genomic loci.

Our data can agree with a “viral mimicry” mechanism for the induction of an anti-viral immune response to endogenous retroelements, which could then increase the efficacy of drug treatments in SCLC [11]. IFI16 and RIG-I were demonstrated to be upregulated in the lung of flu virus-infected mice and IFI16 was shown to positively regulate the anti-viral RIG-I signaling pathway during virus infection [35]. IFI16 can directly bind to RIG-I gene promoter and recruit RNA Pol II, resulting in the upregulation of RIG-I gene and enhancing its activity of sensing viral RNAs [35]. Therefore, the detected positive correlation of LTR30 with RIG-I and IFI16 may be due to a regulatory circuit involving the cytosolic accumulation of ERV-derived transcripts, transcriptional activation of RIG-I by IFI16 and immune gene responses. Notably, recent in vitro data indicate that IFI16, but not cGAS, is able to interact strongly with single-stranded oligos bearing HERV-K LTR sequences corresponding to the first product of reverse transcription in cells and suggesting that the sensor has sequence-specific, yet undetermined, binding patterns [25]. Therefore, IFI16 may also exploit its function of DNA sensor binding to reverse transcribed products of ERV LTR30 and triggering an innate immune response via activation of STING [23]. Thus, our data are consistent with the recognition of LTR30 due to a regulated crosstalk between RNA sensors (mainly RIG-I) with DNA sensors (as IFI16) in SCLC.

The findings also provide unexpected insights into the mechanism of the role of ERV LTR TE expression in the modulation of innate immune responses in human SCLC. TEs are known to function as nucleation centres for facultative heterochromatin [36], as well as binding sites for transcription factors that can affect the expression of adjacent [17] and distal genes [37]. We observed a significant enrichment of H3K4me2 (and other euchromatin marks) at intergenic genomic loci of LTR30 in cultured cancer cells. Interestingly, intergenic LTR30 loci are often close to downregulated genes in SCLCs and overlap with estrogen receptor 1 (ESR1) transcription factor binding sites (Fig. 4). ESR1 is upregulated in NSCLCs favouring cancer survival and progression [38], whereas it is downregulated in SCLC suggesting that ESR1 binding to LTR30 loci is reduced. In addition, the de-methylase LSD1, which targets H3K4me2, is upregulated in SCLC. Interestingly, LSD1 localizes at LTR30 elements, and other downregulated ERV intergenic loci (Fig. 5) in a SCLC cell line model, indicating that LSD1 may be responsible for the loss of a euchromatin marker (H3K4me2) in human SCLC. In contrast to other cancers [10, 14], our data do not support alterations of DNA methylation patterns at intergenic LTR30 loci in SCLC vs normal lung tissues (Fig. S8). Thus, downregulation of LTR30, LTR9C, LTR22C and MER61F subfamilies can be a consequence of tumour specific modifications, such as a chromatin repressive mechanism mediated by LSD1, in order to prevent the recognition of ERV transcripts by cytosolic RNA sensors and the consequent activation of innate immune response. Interestingly, in melanoma cells, histone demethylase LSD1 inhibition increases repetitive element expression and anti-tumour immunity response [39], suggesting that its pharmacological targeting may be a promising strategy to enhance innate immune response in cancers.

In conclusion, we provide strong evidence of the importance of the expression of tumor-repressed ERV LTR subfamilies to activate an innate immune gene response in SCLC tumours. Higher expression levels of LTR30 can predict favourable prognosis in SCLC patients, suggesting a potential role of LTR expression in activating innate immune response that may serve as a promising combined therapeutic approach to be associated with chemotherapy and immunotherapy. Thus, our present findings indicate that the reactivation of patient-specific LTR subfamilies may be a potential strategy for the treatment of immunologically unresponsive SCLC.

## Material and methods

### Data collection

Human SCLC mRNA sequencing data (*fastq* format) and clinical data from the European Genome-Phenome Archive were collected from datasets with the following accession numbers: EGAD00001001244 (*n* = 58, primary tumour samples) [1]*;* EGAD00001001431 (*n* = 15 primary tumour samples) [20]; EGAD00001000223 (*n* = 31 primary tumour samples, *n* = 24 matched normal lung samples) [19]. Each downloaded library was prepared from unstranded, poly(A) selected RNA using Illumina HiSeq 2000 technology and quality checked with FastQC.

### RNA-Seq analysis

Raw RNA-Seq libraries were processed and analysed as described in the mRNA-Seq pipeline (*Dr15plus* version) provided by GDC at https://docs.gdc.cancer.gov, as previously reported [9]. Briefly, adapter trimming and filtering on paired-end RNA-Seq reads were performed using Trimmomatic [40] (version 0.36, RRID:SCR_011848), then reads were aligned to the human reference genome (UCSC hg38, assembly ID GRCh38.p13, Dec.2017) using STAR (version 2.7.10a, RRID:SCR_004463) [41] and SAMtools (version 1.15, RRID:SCR_002105) was used for sorting and indexing the aligned BAM file [42]. HT-Seq (version 0.13.5. RRID:SCR_011867) [43] was used for the quantification of mapped reads to each gene. To annotate genes, we used the *getBM* function from the “bioMart” package (version 2.50.3) in R to retrieve the HGNC symbols from the Ensembl database (release 104) associated to the gene Ensembl IDs filtered by the gene name IDs from GENCODEv22 as input values. Genome index for alignment step and gene reference annotation (GENCODE v22) were obtained from GDC reference files portal. Then, aggregation of gene counts and downstream analyses were performed using R.

### Quantification of TE expression and differential expression analysis

Quantification of TE expression in SCLC samples was performed using *REdiscoverTE*, a software that allows a whole-transcriptome RNA-Seq quantification simultaneously for repetitive sequences and transcriptome [14]. Quantification output files of *REdiscoverTE* are DGEList data type in the. RDS R data format, which requires “edgeR” library (version 3.14.0, RRID:SCR_012802) to be read in R. After quantification, intergenic raw counts were used for downstream analyses. A differential expression analysis was performed using the Bioconductor (RRID:SCR_006442) package “DESeq2” (version 1.34.0, RRID:SCR_000154) [44]. A DESeqDataSet from the matrix of intergenic TEs raw counts for SCLC tumour and matched normal samples (*n* = 48) was built considering the type of tissues (tumour or normal) and the variability among patients, in the *design* parameter. Pre-filtering for counts ≥5 was performed before counts normalization. Results from DESeq2 normalization were extracted setting the parameters *alpha* = 0.05, *lfcThreshold* = 0.58 and *altHypothesis* = “*greaterAbs*” and *shrinkage* of log2 fold change was then performed. Downstream analyses concerned only TE subfamilies belonging to LINE (long interspersed element), SINE (short-interspersed element), LTR (long-terminal repeat), Retroposon (SVA) and DNA transposon classes. TE subfamilies with cut-off of the absolute maximum a posteriori fold-change ≥1 and P_adj_ ≤ 0.05 were considered as differentially expressed. Volcano plot showing differential expression of TEs was made in R using the “EnhancedVolcano” Bioconductor package (version 1.12.0, RRID:SCR_018931). PCA (Principal Component Analysis) plot was made using *plotPCA* function from “DESeq2” R package on *variance stabilizing transformation* of counts.

### Locus-level quantification of TE expression

Raw reads for each RNA-seq library were trimmed and filtered as in “RNA-Seq analysis” section and aligned using the same version of STAR with flags that allow multimapped reads retention. Quantification of locus-level TE expression was performed with TElocal (v 1.1.1 ) [21] tool (https://github.com/mhammell-laboratory/TElocal) using available default index and settings, considering all genomic loci.

### Gene set variation analysis (GSVA) and Spearman correlation

To obtain enrichment scores for the estimation of pathway activity variation over the tumour sample population, a gene set variation analysis was performed in R using the *gsva* function from the “GSVA” Bioconductor package (version 1.42.0, RRID:SCR_021058) [45], with “*gsva*” method parameter, and the Molecular Signature Database gene sets from the Hallmark collection (version 7.5.1, RRID:SCR_016863) [46], as well as immune-related gene sets from Kong et al., where the “Response to type I Interferon” gene set has been implemented with the “Response to type I interferon” signature from GO Biological Processes. Correlation between TEs expression and enrichment scores for gene sets was performed with *cor.test* function in stats package (version 4.1.3) to obtain Spearman’s *rho* correlation coefficient and *p*-values. Heatmaps of correlation were made using the R package “ComplexHeatmap” (RRID:SCR_017270) [47]. Scatter plots and violin plots were made using “ggplot2” (version 3.3.5, RRID:SCR_014601) and “ggpubr” (version 0.4.0, RRID:SCR_021139) R packages.

### Genomic locus overlap enrichment analysis

Genomic locus overlap enrichment analysis was performed using LOLA R package (version 1.24.0) [27]. Testing for overlaps of TEs genomic regions with publicly available databases of genomic range sets were performed using transcription factor binding sites and histone marks Epigenome database from Cistrome [28], included in LOLA Core database (hg38) at http://big.databio.org/regiondb/. TE genomic coordinates were downloaded from UCSC Genome Browser in a BED file format. Bubble plot for the genomic locus overlap enrichment analysis was made using “ggplot2” R package. LOLA was run for all deregulated LTRs using all intergenic LTRs genomic loci as “universe”. Motif discovery analysis for each TE candidate was performed using Homer tool (v4.11) [48] with default settings and using a dataset random background sequences matched for GC% content of the target sequences.

### Gene set enrichment analysis (GSEA) for genes proximal to TEs

To evaluate the potential influence of differential expressed TEs activity on adjacent genes expression, genomic coordinates for genes and TEs were downloaded from UCSC Genome Browser in a BED file format, then we considered the list of genes with a TSS (Transcription Start Site) up to 20 kb downstream from the genomic coordinates of each specific TE as input for the gene set enrichment analysis [49]. The GSEA was performed using the *GSEA* function from the “clusterProfiler” R package (version 4.2.2) [50]*,* with default options. GSEA plot was performed using the *GSEAplot* function and the ridgeline plots of the GSEA results were made by *ridgeplot* function from “clusterProfiler” package (RRID:SCR_016884).

### Methylation data analysis and H3K4me2/LSD1 occupancy

Methylation data were kindly obtained from Poirier et al. [32] upon request. For each selected LTR, we selected 5′-CpG dinucleotides based on their localization in gene body or 1000 bp upstream or downstream the element. Plots were made using “ggplot” and “ggpubr” R libraries. H3K4me2 and LSD1 ChIP-seq and relative input genomic signal data were downloaded as processed bigWig from GEO omnibus (GSE66297) [31]. Intergenic genomic loci for LTR30, LTR22C, LTR9C and MER61F were downloaded from UCSC table browser. Signal plot were produced using deeptools2 (RRID:SCR_016366) *computeMatrix* and *plotProfile* commands [51].

### Survival analysis

Survival analysis was performed using the clinical data available for SCLC patients (*n* = 73), first filtering samples from patients who did not receive previous treatments for SCLC before surgical resection of the tumour and further considering the availability of information regarding the overall survival, the status at last time of follow-up and chemotherapy treatment. We also considered the expression levels of ERVs as a variable to perform the estimates, grouping samples with “high” expression levels (> 75th percentile) and “low” expression levels (< 25th percentile). We used “survival” package to compute a multi-variate analysis of survival estimates and “survminer” package (RRID:SCR_021094) to obtain a Kaplan-Meier plot of the results.

## Supplementary Information

**Additional file 1: Fig. S1. A)** Fraction of intergenic (blue), exonic (yellow) and intronic (orange) TE subfamilies expression grouped by classes over the total expression levels. **B)** PCA plot of SCLC/normal lung datasets based on gene expression. Samples are tumour (magenta triangles) and normal lung (green circles) samples. **C)** Heatmap of deregulated TE normalized counts of tumour and matched normal samples expressed as row z-score, colour as in legend. Clusters by row are according to patient ID, while clusters by column are according to upregulation or downregulation of TE in tumour samples respect with matched normal. Log_2_FC annotation: log_2_ fold change values of differentially expressed TEs, colour as in legend. Log_2_ Mean Expression annotation: line plot with dots corresponding to the log_2_ mean expression for each differentially expressed TE, colour as in legend. Type annotation: Sample tissue type, colour as in legend. **D)** Heatmap of tumour vs matched normal samples normalized counts ratio (rows) for each differentially expressed TEs (columns), colour as in legend. Clusters by column are according to upregulation or downregulation of TE in tumour samples respect with matched normal. Log_2_FC annotation: log_2_ fold change values of differentially expressed TEs, ranked from the negative (yellow) to the positive (green) values, as in legend. **Fig. S2. A)** Heatmap of correlation between expression levels of differentially-expressed TEs (rows) and MsigDB Hallmark collection signatures GSEA scores (columns). Heatmap colour: Spearman’s *rho* correlation coefficient (Cor). Log_2_FC annotation: log_2_ fold change values of differentially expressed TEs, ranked from the negative (yellow) to the positive (green) values, as in legend. Class annotation: TE Class, colour as in legend. Interferon responses and inflammatory signatures are highlighted by green box. *p*-values: * 0.001-0.05, ** 0.00001-0.001, *** < 0.00001. None, NS. **Fig. S3. A)** Fraction of intergenic (blue), exonic (yellow) and intronic (orange) LTR22C, LTR30, LTR9C and MER61F expression over the total expression levels. Number of loci for each genomic region is reported for each subfamily. **Fig. S4.** Volcano plot of differentially-expressed loci in 24 SCLC vs matched normal samples for **A)** LTR30, **B)** LTR9C, **C)** LTR22C, **D)** MER61F, **E)** HERVL1_LTRd and **F)** MER74C subfamilies. X-axis: log2 fold change values of differential expression. Y axis: -Log10(q-value). Points correspond to TE loci. Red points correspond to differentially-expressed TE loci with log2 fold change ≥ + 1 or log2 fold change ≤ − 1 and q-value ≤0.05. Yellow points correspond to differentially expressed TE loci with log2 fold change ≥ + 1 or log2 fold change ≤ − 1 and q-value ≥0.05. **Fig. S5. A)** Heatmap of correlation between expressed loci of LTR30, LTR22C, LTR9C, MER61F and HERV1_LTRd subfamilies expression (rows) and ssGSEA scores of immune response genes (columns) in SCLC tumour samples. Heatmap colour: Spearman’s *rho* correlation coefficient (Cor). Log_2_FC annotation: log_2_ fold change values of differentially expressed loci in tumour vs normal samples, ranked from the negative (yellow) to the positive (green) values, as in legend. Asterisks in Log_2_FC annotation indicate loci with a significant (*p*-adjusted ≤0.1) differential expression between tumour and normal samples. Mean annotation: mean count expression of loci. *p*-values in heatmap cells: * 0.001-0.05, ** 0.00001-0.001, *** < 0.00001. None, NS. **Fig. S6. A-I)** Scatter plot of correlation between log_2_ normalized counts of cytosolic and endosomal nucleic acids sensors expression (x-axis) and Response to type I Interferon ssGSEA scores (y-axis). Points correspond to SCLC tumour samples (*n* = 104). Colour: Response to type I Interferon ssGSEA scores, as in legend. Spearman’s *rho* (R) and *p*-values of correlation are reported. **Fig. S7. A)** PCA plots of SCLC tumour samples based on cytosolic/endosomal nucleic acids sensors and LTR9C, LTR22C and MER61F expression, separately. Points correspond to SCLC tumour samples (*n* = 104). PCA loadings describing the contribution to the components of each gene and TE are represented as labelled arrows. Colour: Response to type I Interferon ssGSEA scores, as in legend. **Fig. S8. A-D)** Main: Scatter plot of correlation between log_2_ normalized counts of LTR30 expression (x-axis) and log_2_ normalized counts of cytosolic and endosomal nucleic acids sensors expression (y-axis). Points correspond to SCLC tumour samples (*n* = 104). Colour: Response to type I Interferon ssGSEA scores, as in legend. Spearman’s *rho* (R) and *p*-values of correlation are reported. Down: Violin plot of LTR30 expression in samples which Response to type I Interferon is downregulated (blue, NES < − 0.2) or upregulated (red, NES > 0.2). Left: Violin plot of cytosolic and endosomal nucleic acids sensors expression in samples which Response to type I Interferon is downregulated (blue, NES < − 0.2) or upregulated (red, NES > 0.2). Wilcoxon test *p*-values are reported: * 0.01-0.05, ** 0.0001-0.01, *** 0,00001-0.0001, **** < 0,000001. **Fig. S9. A)** Scatter plot of correlation between -Log(*p*-value) (*y*-axis) and “support” percentage, expressed as the ratio between the number of intergenic LTR30 loci overlapping with Cistrome marks loci and the totality of intergenic LTRs loci (*x*-axis). Colours and labels: Cistrome marks, as in legend. **Fig. S10.** Bubble plot of top ten ranked Cistrome Epigenome marks associated to **A)** downregulated LTR elements in tumour samples respect with matched normal and **B)** upregulated LTR elements in tumour samples respect with matched normal. Bubble colour: -Log(*p*-value), as in legend. Bubble size: “support” percentage, expressed as the ratio between the number of intergenic DE LTRs loci overlapping with Cistrome markers loci and the totality of intergenic LTRs loci. **Fig. S11.** Bubble plot of top ranked Cistrome TFBSs associated to **A)** downregulated LTR elements in tumour samples respect with matched normal and **B)** upregulated LTR elements in tumour samples respect with matched normal. Bubble colour: -Log(*p*-value), as in legend. Bubble size: “support” percentage, expressed as the ratio between the number of intergenic DE LTRs loci overlapping with Cistrome markers loci and the totality of intergenic LTRs loci. **Fig. S12** Homer de novo motif results for LTR30 (default output). Results are ranked by *p*-value. **Fig. S13** Homer de novo motif results for MER61F (default output). Results are ranked by *p*-value. **Fig. S14** Homer de novo motif results for LTR22C (default output). Results are ranked by *p*-value. Asterisks in “Rank” field indicate potential false positives. **Fig. S15. A)** Mean normalized read levels for H3K4me2 over downregulated expressed ERVs at locus level, in H526 SCLC cell line **B)** Mean genomic signal of ChIP/input ratio over downregulated expressed ERVs at locus level for LSD1 in H526 SCLC cell line in untreated (blue) and treated with LSD1 inhibitor (green) conditions. **Fig. S16. A-C)** Methylation level (left panel) in SCLC tumours (blue) compared to matched normal samples (red) in cg located in 1000 bp upstream **(A)**, genebody **(B)** and 1000 bp downstream **(C)** of LTR30 loci. For each cg, correlation between methylation level and LTR30 expression is reported on right panel. **D)** Correlation between methylation levels (y-axis) and STING expression (x-axis) in SCLC tumours for cg located upstream (cg16983159 and cg23255964) and in the gene body (cg04232128) of STING gene. **Fig. S17 A-C)** Kaplan-Meier plot of survival estimates of SCLC patients’ subgroups (total *n* = 30) depending on expression levels of LTR22C (**A**) MER61F (**B**) LTR9C (**C**) and chemotherapeutic treatments. Log rank test *p*-values are reported. Curves colours are as in legend.

**Additional file 2: TableS1.** Table of differentially expressed intergenic TE subfamilies (REdiscoverTE).

**Additional file 3: TableS2.** Table of differentially expressed global TE subfamilies (REdiscoverTE).

**Additional file 4: TableS3.** Table of differentially expressed LTR30, LTR22C, LTR9C, MER61F and HERV1_LTRd subfamilies at locus level (TElocal).

## Data Availability

Human SCLC mRNA sequencing data (*fastq* format) and clinical data from the European Genome-Phenome Archive were collected from datasets with the following accession numbers: EGAD00001001244 (*n* = 58, primary tumour samples) [1]*;* EGAD00001001431 (*n* = 15 primary tumour samples) [20]; EGAD00001000223 (*n* = 31 primary tumour samples, *n* = 24 matched normal lung samples) [19]. Methylation data were kindly obtained from Poirier et al. [32] upon request. All the code used to produce data and figures is available at: https://github.com/marcrusso/TE_SCLC_2022.

## Abbreviations

DDR: DNA damage response
DE: Differentially expressed
ERV: Endogenous retrovirus
LINE: Long-interspersed element
LTR: Long-terminal repeat
PCA: Principal component analysis
SCLC: Small-cell lung cance
SINE: Short-interspersed element
TE: Transposable elements
TFBS: Transcription factor binding site
