# Supplementary material for: Expression of down-regulated ERV LTR elements associates with immune activation in human small-cell lung cancers
Source: Mob DNA. 2023 Mar 14;14:2. doi: 10.1186/s13100-023-00290-w (PMC10012523; doi:10.1186/s13100-023-00290-w)
Supplement: Supplementary file 1 — Additional file 1: Fig. S1. A) Fraction of intergenic (blue), exonic (yellow) and intronic (orange) TE subfamilies expression grouped by classes over the total expression levels. B) PCA plot of SCLC/normal lung datasets based on gene expression. Samples are tumour (magenta triangles) and normal lung (green circles) samples. C) Heatmap of deregulated TE normalized counts of tumour and matched normal samples expressed as row z-score, colour as in legend. Clusters by row are according to patient ID, while clusters by column are according to upregulation or downregulation of TE in tumour samples respect with matched normal. Log2FC annotation: log2 fold change values of differentially expressed TEs, colour as in legend. Log2 Mean Expression annotation: line plot with dots corresponding to the log2 mean expression for each differentially expressed TE, colour as in legend. Type annotation: Sample tissue type, colour as in legend. D) Heatmap of tumour vs matched normal samples normalized counts ratio (rows) for each differentially expressed TEs (columns), colour as in legend. Clusters by column are according to upregulation or downregulation of TE in tumour samples respect with matched normal. Log2FC annotation: log2 fold change values of differentially expressed TEs, ranked from the negative (yellow) to the positive (green) values, as in legend. Fig. S2. A) Heatmap of correlation between expression levels of differentially-expressed TEs (rows) and MsigDB Hallmark collection signatures GSEA scores (columns). Heatmap colour: Spearman’s rho correlation coefficient (Cor). Log2FC annotation: log2 fold change values of differentially expressed TEs, ranked from the negative (yellow) to the positive (green) values, as in legend. Class annotation: TE Class, colour as in legend. Interferon responses and inflammatory signatures are highlighted by green box. p-values: * 0.001-0.05, ** 0.00001-0.001, *** < 0.00001. None, NS. Fig. S3. A) Fraction of intergenic (blue), exonic (yellow) an [file 13100_2023_290_MOESM1_ESM.docx]

**Supplementary file.**

**Expression of down-regulated ERV LTR elements associates with immune activation in human small-cell lung cancers.**

Marco Russo*, Sara Morelli* and Giovanni Capranico.

*Department of Pharmacy and Biotechnology, Alma Mater Studiorum – University of Bologna, via Selmi 3, 40126 Bologna, Italy*

*Supplementary tables description*

*TableS1: Table of differentially expressed intergenic TE subfamilies (REdiscoverTE).*

*TableS2: Table of differentially expressed global TE subfamilies (REdiscoverTE).*

*TableS3: Table of differentially expressed LTR30, LTR22C, LTR9C, MER61F and HERV1_LTRd subfamilies at locus level (TElocal)*


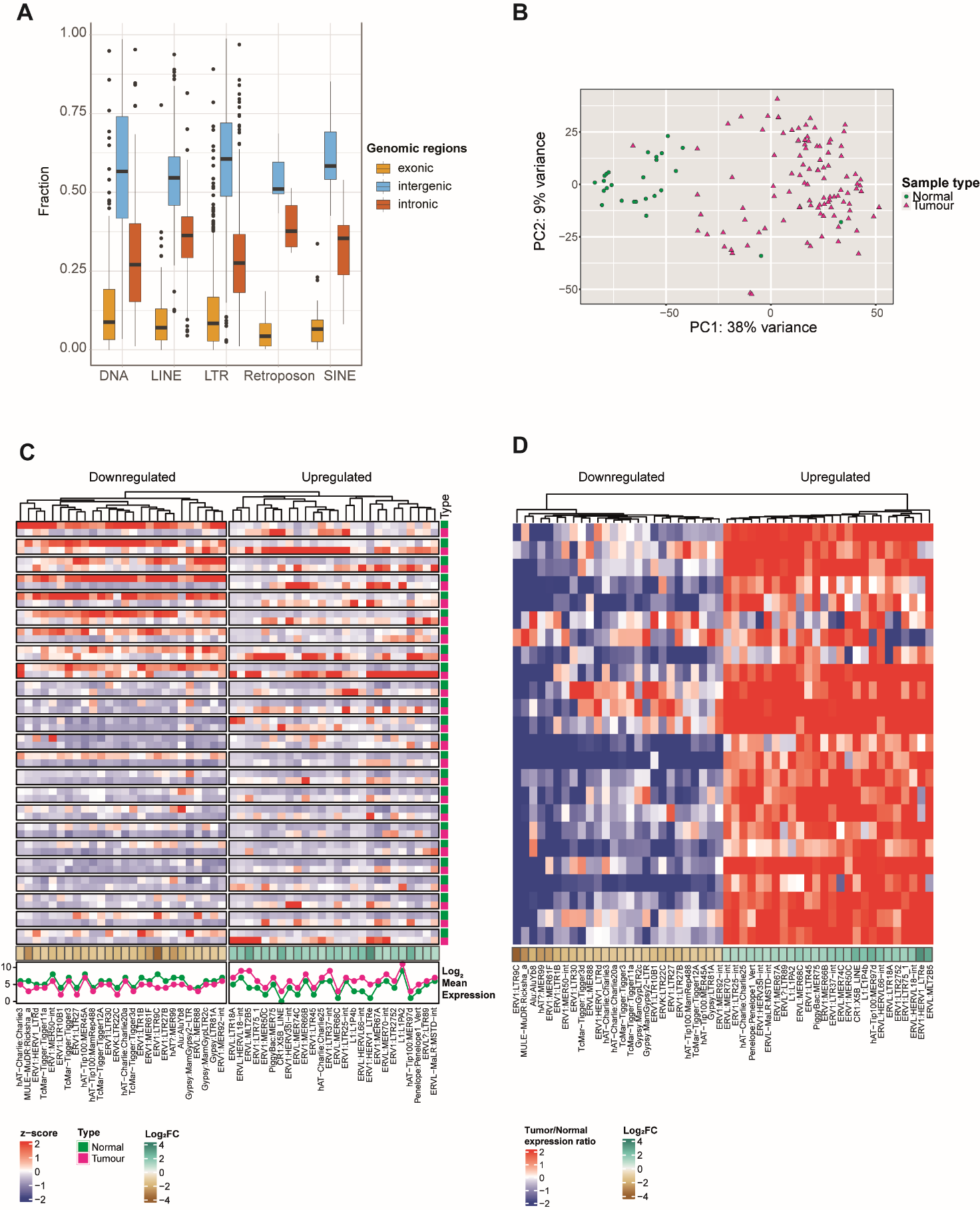


**Figure S1. A)** Fraction of intergenic (blue), exonic (yellow) and intronic (orange) TE subfamilies expression grouped by classes over the total expression levels. **B)** PCA plot of SCLC/normal lung datasets based on gene expression. Samples are tumour (magenta triangles) and normal lung (green circles) samples. **C)** Heatmap of deregulated TE normalized counts of tumour and matched normal samples expressed as row z-score, colour as in legend. Clusters by row are according to patient ID, while clusters by column are according to upregulation or downregulation of TE in tumour samples respect with matched normal. Log_2_FC annotation: log_2_ fold change values of differentially expressed TEs, colour as in legend. Log_2_ Mean Expression annotation: line plot with dots corresponding to the log_2_ mean expression for each differentially expressed TE, colour as in legend. Type annotation: Sample tissue type, colour as in legend. **D)** Heatmap of tumour vs matched normal samples normalized counts ratio (rows) for each differentially expressed TEs (columns), colour as in legend. Clusters by column are according to upregulation or downregulation of TE in tumour samples respect with matched normal. Log_2_FC annotation: log_2_ fold change values of differentially expressed TEs, ranked from the negative (yellow) to the positive (green) values, as in legend.


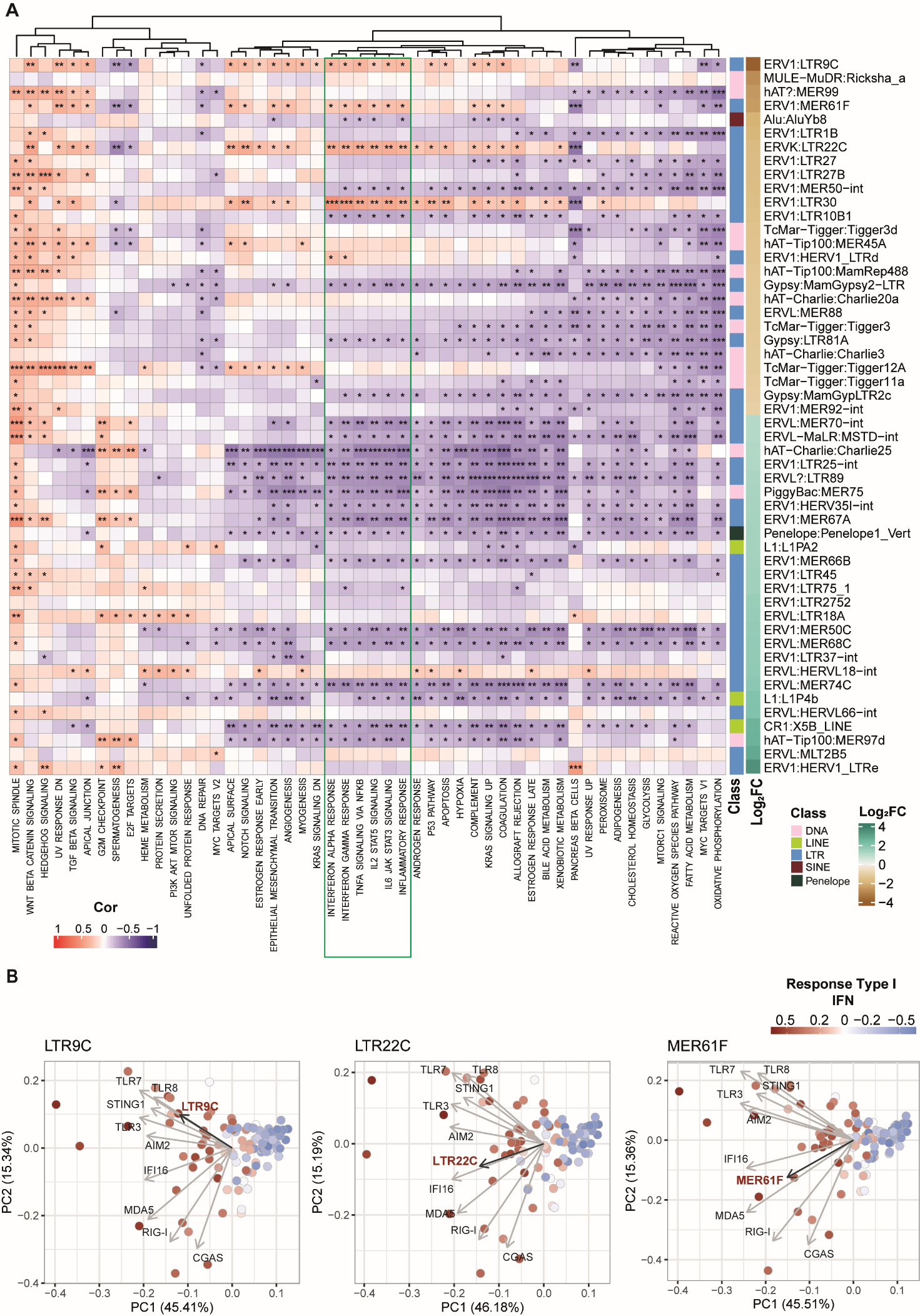


**Figure S2. A)** Heatmap of correlation between expression levels of differentially-expressed TEs (rows) and MsigDB Hallmark collection signatures GSEA scores (columns). Heatmap colour: Spearman’s *rho* correlation coefficient (Cor). Log_2_FC annotation: log_2_ fold change values of differentially expressed TEs, ranked from the negative (yellow) to the positive (green) values, as in legend. Class annotation: TE Class, colour as in legend. Interferon responses and inflammatory signatures are highlighted by green box. *p*-values: * 0.001-0.05, ** 0.00001-0.001, *** < 0.00001. None, NS.


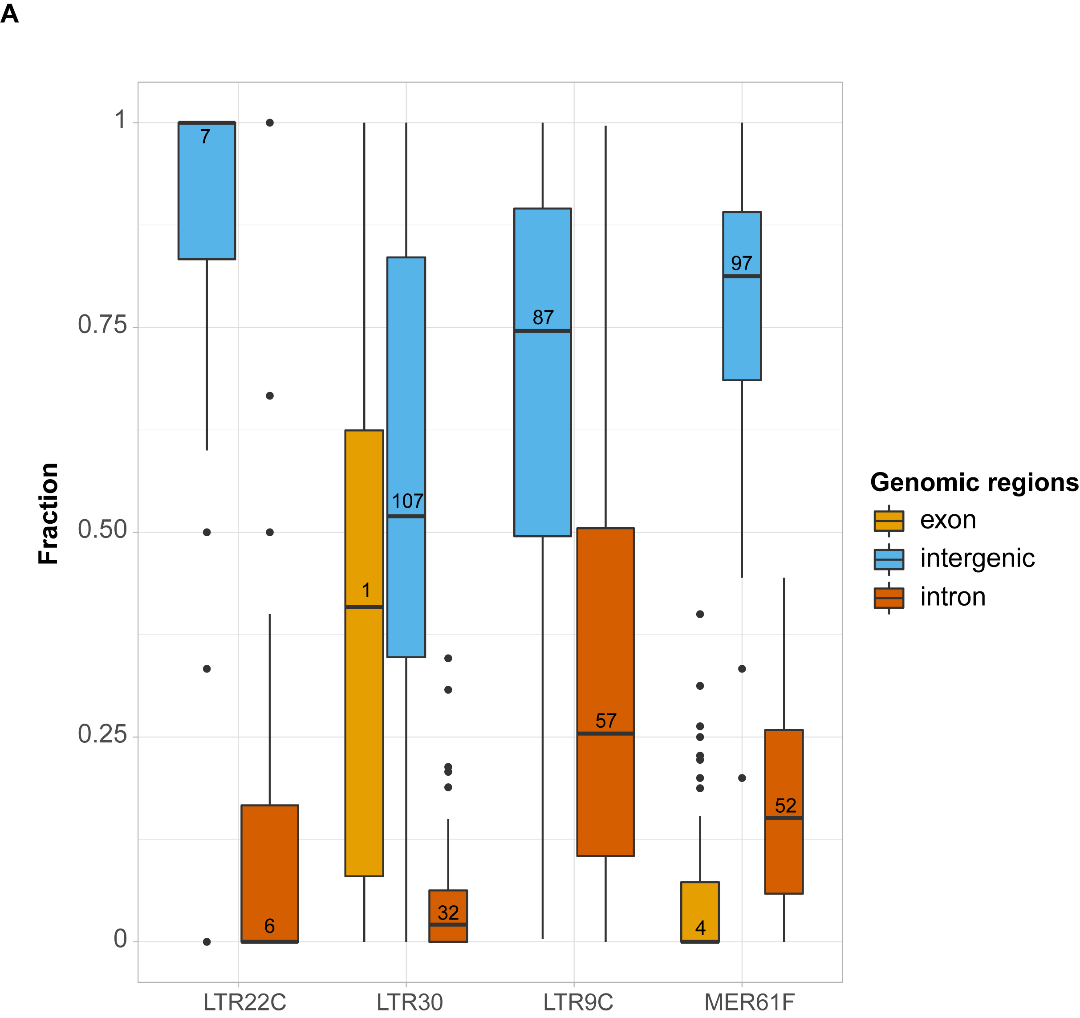


**Figure S3. A)** Fraction of intergenic (blue), exonic (yellow) and intronic (orange) LTR22C, LTR30, LTR9C and MER61F expression over the total expression levels. Number of loci for each genomic region is reported for each subfamily.


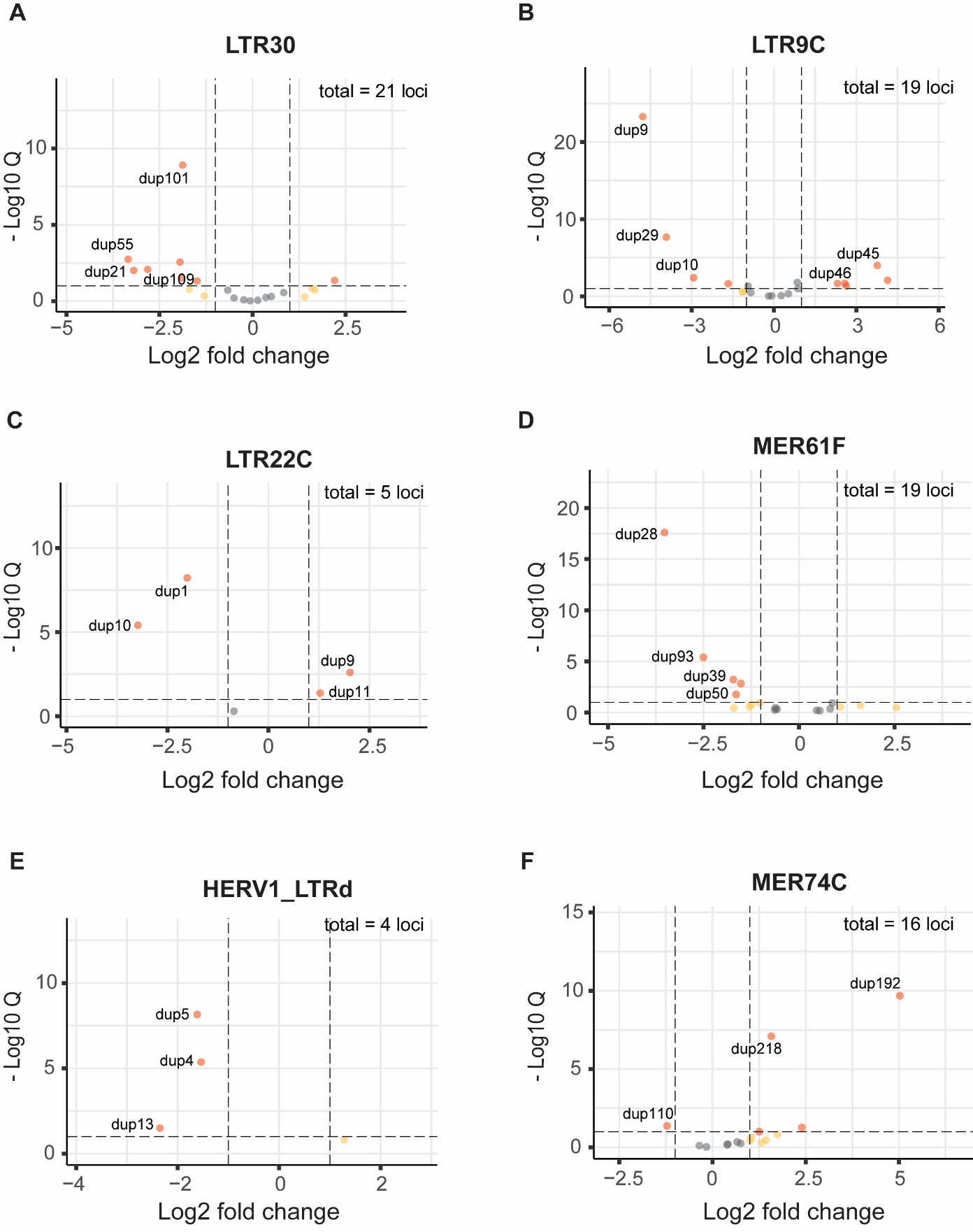


**Figure S4.** Volcano plot of differentially-expressed loci in 24 SCLC vs matched normal samples for **A)** LTR30, **B)** LTR9C, **C)** LTR22C, **D)** MER61F, **E)** HERVL1_LTRd and **F)** MER74C subfamilies. X-axis: log2 fold change values of differential expression. Y axis: -Log10(q-value). Points correspond to TE loci. Red points correspond to differentially-expressed TE loci with log2 fold change ≥ +1 or log2 fold change ≤ -1 and q-value ≤ 0.05. Yellow points correspond to differentially expressed TE loci with log2 fold change ≥ +1 or log2 fold change ≤ -1 and q-value ≥ 0.05.


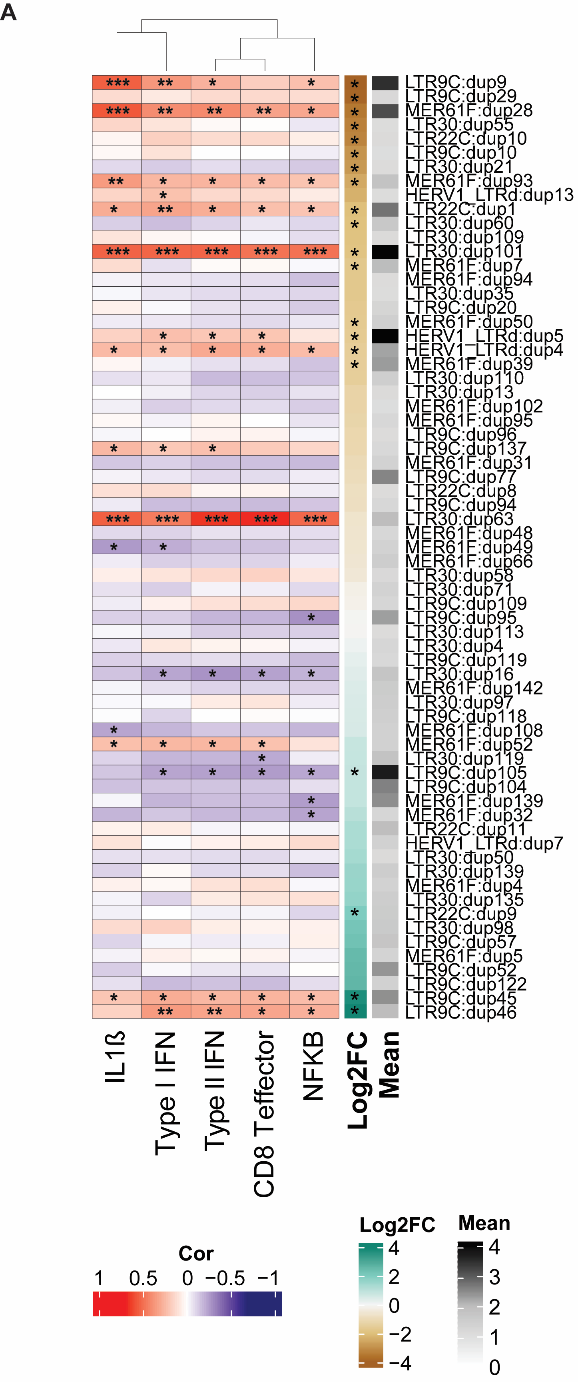


**Figure S5. A)** Heatmap of correlation between expressed loci of LTR30, LTR22C, LTR9C, MER61F and HERV1_LTRd subfamilies expression (rows) and ssGSEA scores of immune response genes (columns) in SCLC tumour samples. Heatmap colour: Spearman’s *rho* correlation coefficient (Cor). Log_2_FC annotation: log_2_ fold change values of differentially expressed loci in tumour *vs* normal samples, ranked from the negative (yellow) to the positive (green) values, as in legend. Asterisks in Log_2_FC annotation indicate loci with a significant (*p*-adjusted ≤ 0.1) differential expression between tumour and normal samples. Mean annotation: mean count expression of loci. *p*-values in heatmap cells: * 0.001-0.05, ** 0.00001-0.001, *** < 0.00001. None, NS.


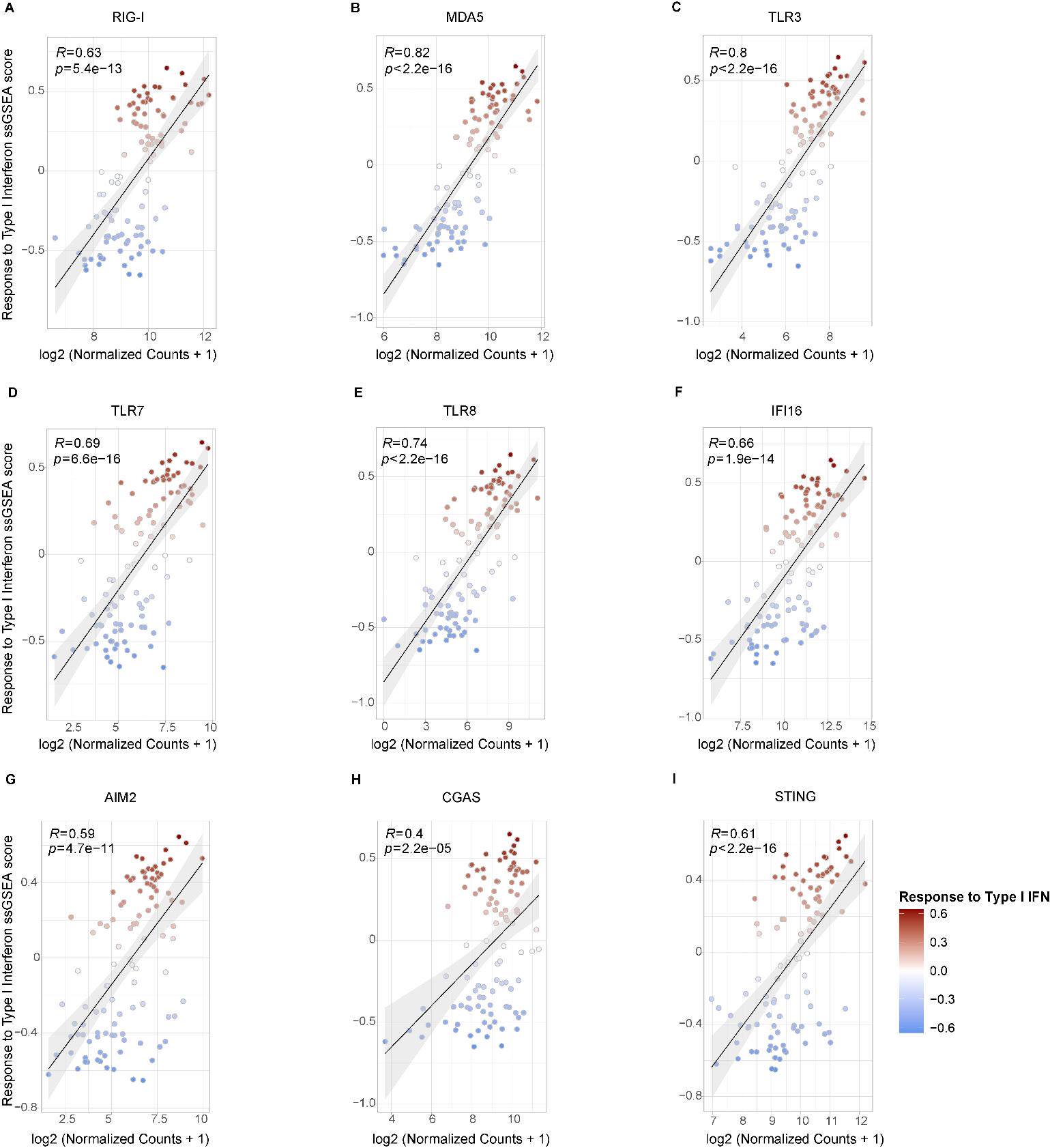


**Figure S6. A-I)** Scatter plot of correlation between log_2_ normalized counts of cytosolic and endosomal nucleic acids sensors expression (x-axis) and Response to type I Interferon ssGSEA scores (y-axis). Points correspond to SCLC tumour samples (*n* = 104). Colour: Response to type I Interferon ssGSEA scores, as in legend. Spearman’s *rho* (R) and *p*-values of correlation are reported.


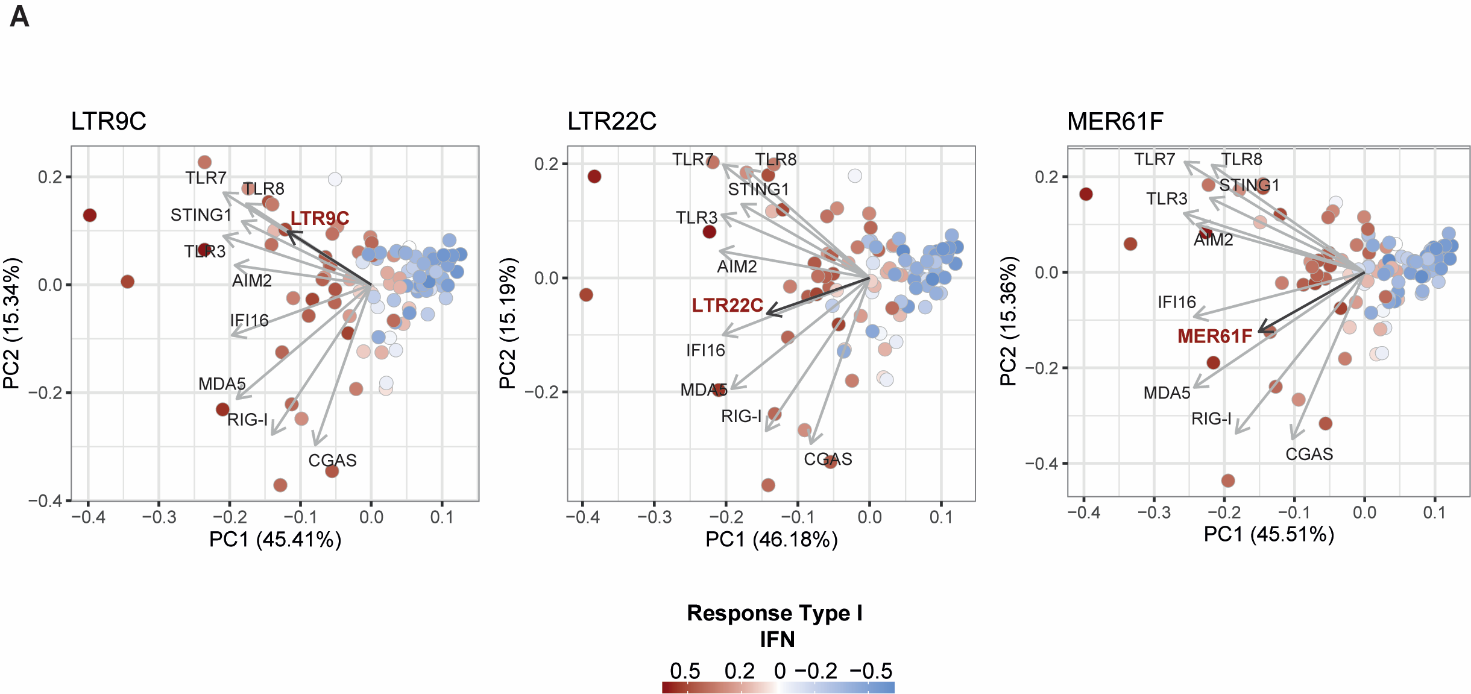


**Figure S7. A)** PCA plots of SCLC tumour samples based on cytosolic/endosomal nucleic acids sensors and LTR9C, LTR22C and MER61F expression, separately. Points correspond to SCLC tumour samples (*n* = 104). PCA loadings describing the contribution to the components of each gene and TE are represented as labelled arrows. Colour: Response to type I Interferon ssGSEA scores, as in legend.


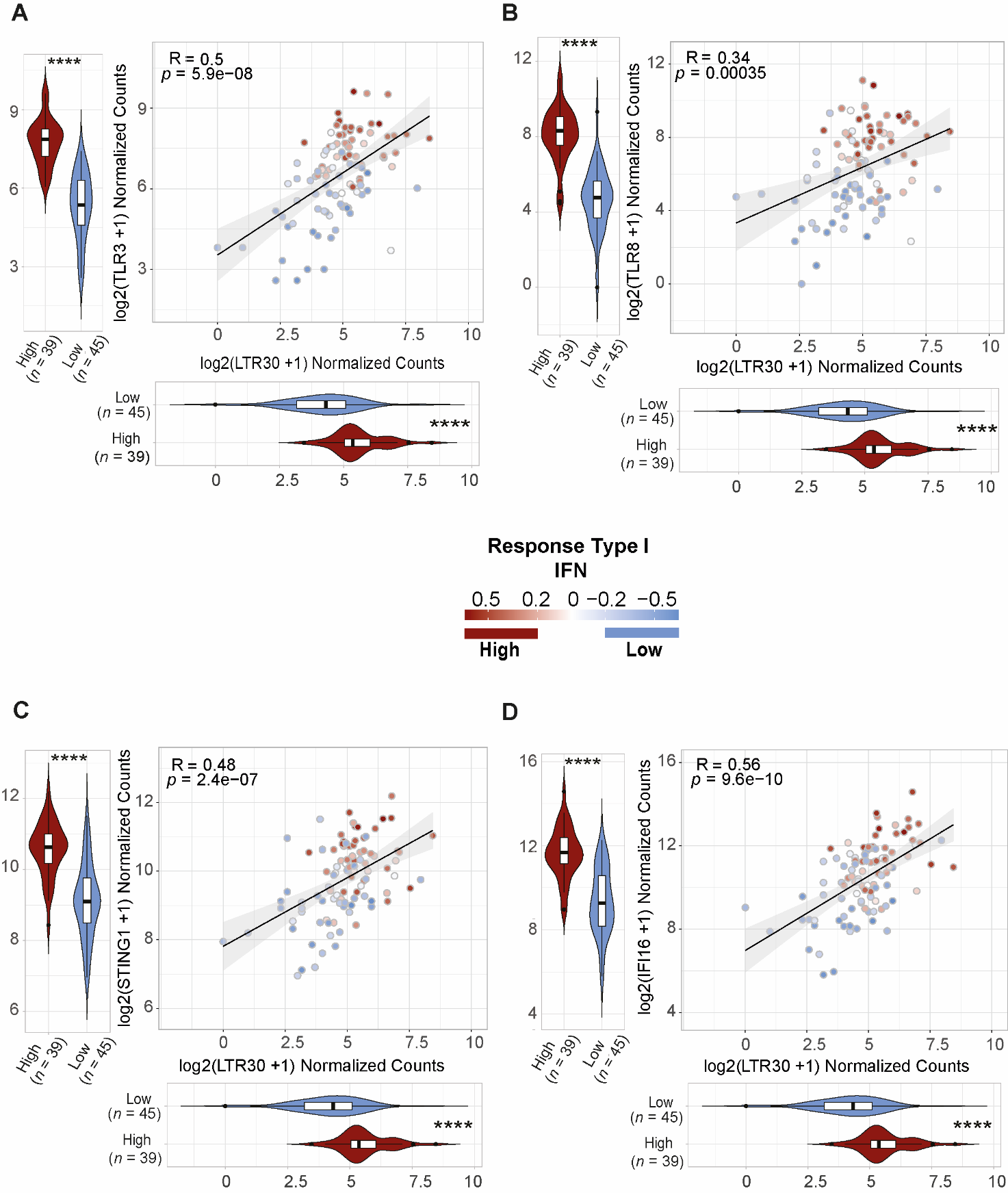


**Figure S8. A-D)** Main: Scatter plot of correlation between log_2_ normalized counts of LTR30 expression (x-axis) and log_2_ normalized counts of cytosolic and endosomal nucleic acids sensors expression (y-axis). Points correspond to SCLC tumour samples (*n* = 104). Colour: Response to type I Interferon ssGSEA scores, as in legend. Spearman’s *rho* (R) and *p*-values of correlation are reported. Down: Violin plot of LTR30 expression in samples which Response to type I Interferon is downregulated (blue, NES < -0.2) or upregulated (red, NES > 0.2). Left: Violin plot of cytosolic and endosomal nucleic acids sensors expression in samples which Response to type I Interferon is downregulated (blue, NES < -0.2) or upregulated (red, NES > 0.2). Wilcoxon test *p*-values are reported: * 0.01-0.05, ** 0.0001-0.01, *** 0,00001-0.0001, **** < 0,000001.


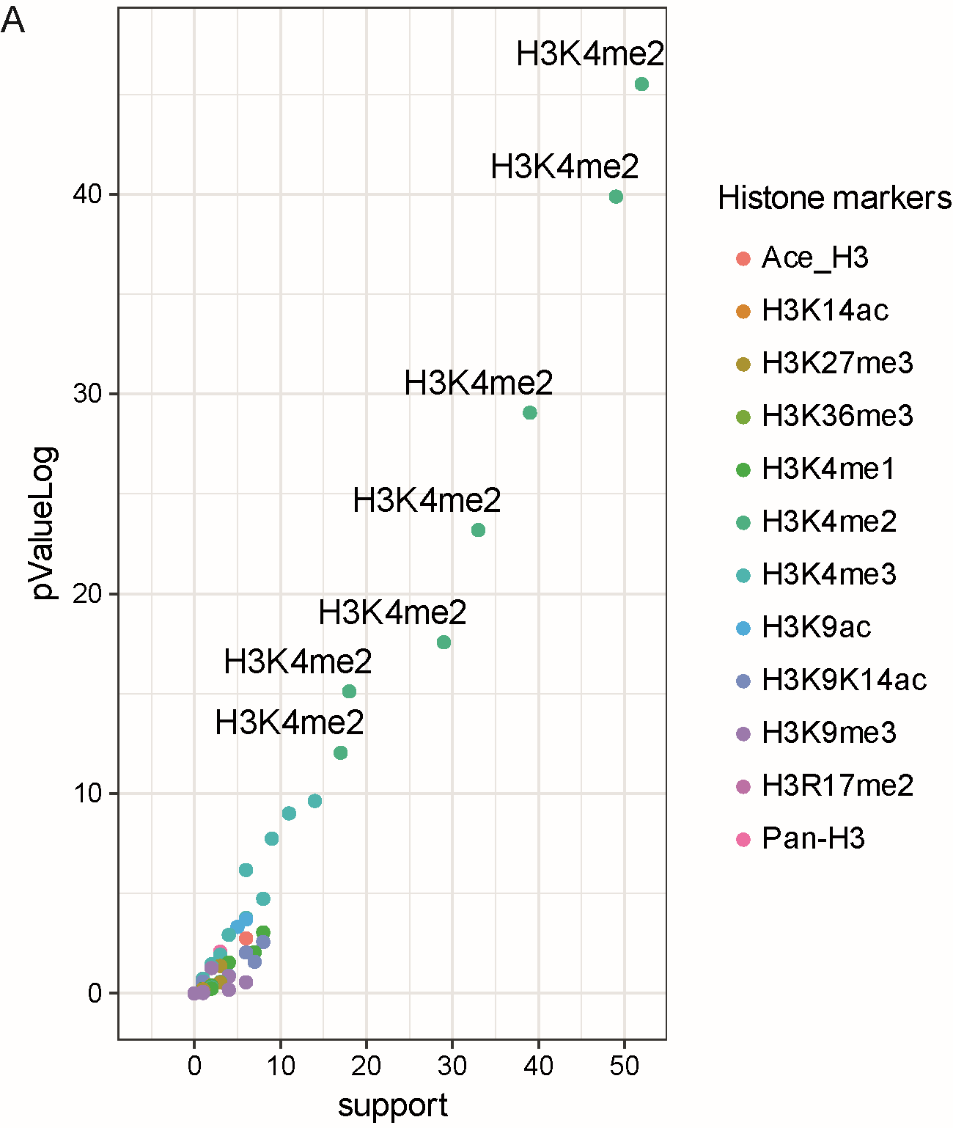


**Figure S9. A)** Scatter plot of correlation between -Log(*p*-value) (*y*-axis) and “support” percentage, expressed as the ratio between the number of intergenic LTR30 loci overlapping with Cistrome marks loci and the totality of intergenic LTRs loci (*x*-axis). Colours and labels: Cistrome marks, as in legend.


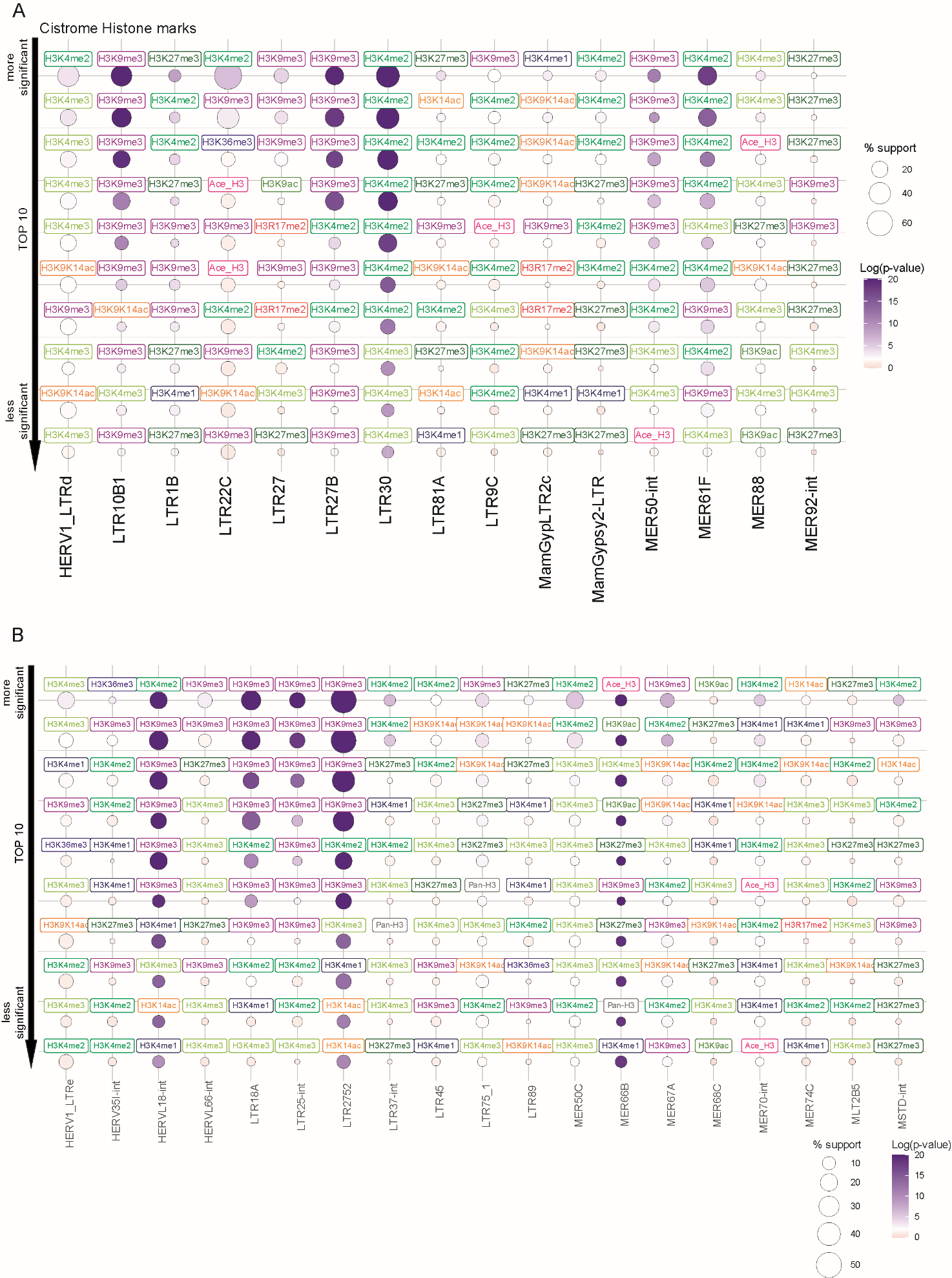


**Figure S10.** Bubble plot of top ten ranked Cistrome Epigenome marks associated to **A)** downregulated LTR elements in tumour samples respect with matched normal and **B)** upregulated LTR elements in tumour samples respect with matched normal. Bubble colour: -Log(*p*-value), as in legend. Bubble size: “support” percentage, expressed as the ratio between the number of intergenic DE LTRs loci overlapping with Cistrome markers loci and the totality of intergenic LTRs loci.


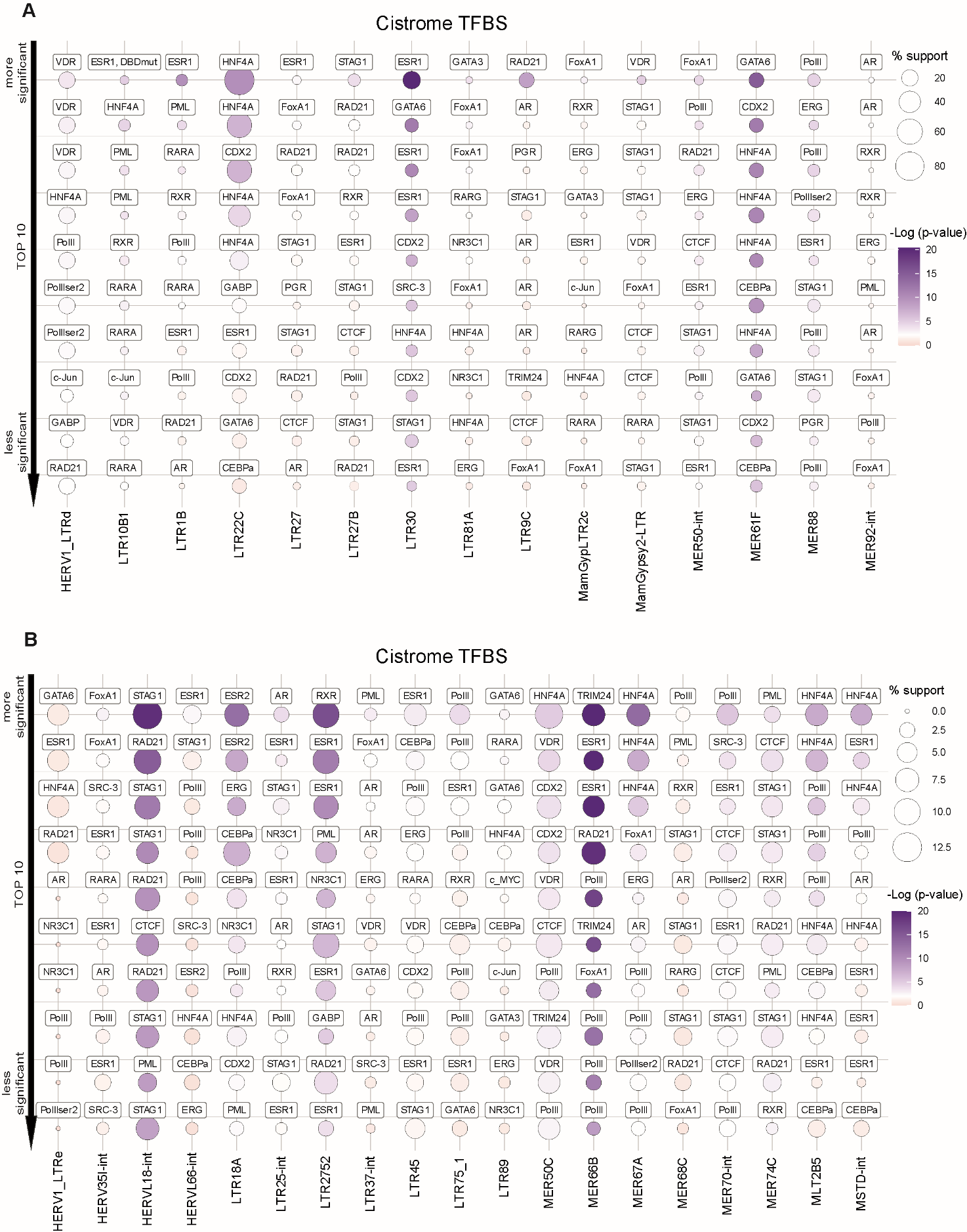


**Figure S11.** Bubble plot of top ranked Cistrome TFBSs associated to **A)** downregulated LTR elements in tumour samples respect with matched normal and **B)** upregulated LTR elements in tumour samples respect with matched normal. Bubble colour: -Log(*p*-value), as in legend. Bubble size: “support” percentage, expressed as the ratio between the number of intergenic DE LTRs loci overlapping with Cistrome markers loci and the totality of intergenic LTRs loci.


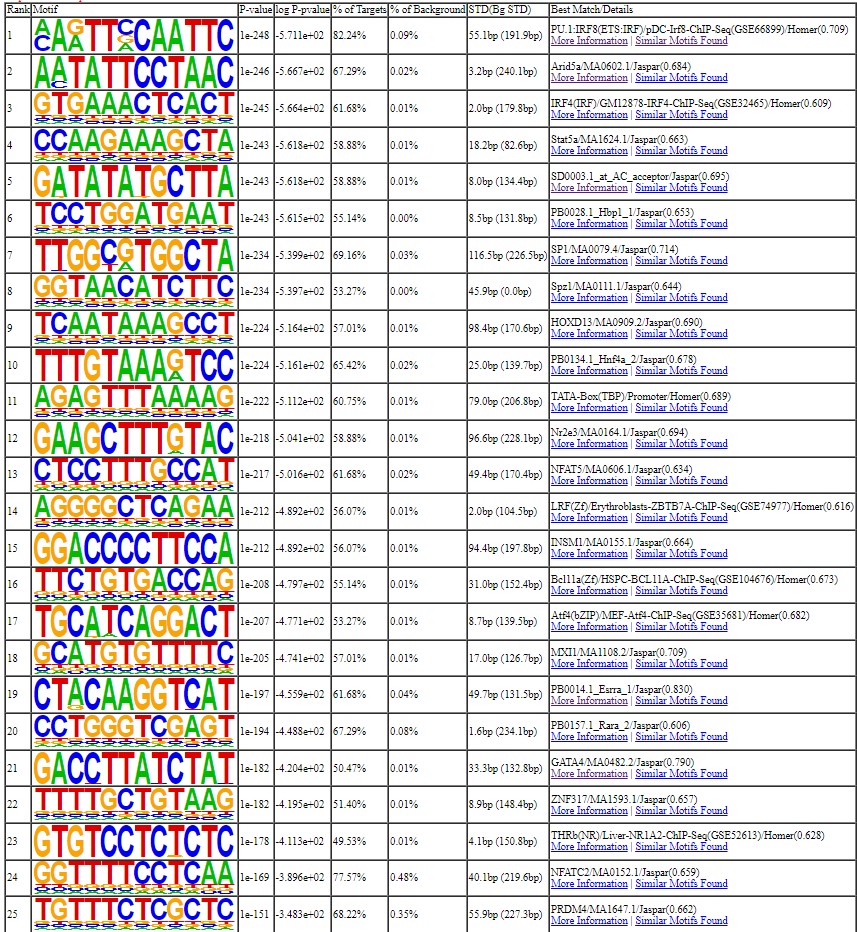


**Figure S12** Homer *de novo* motif results for LTR30 (default output). Results are ranked by *p*-value.


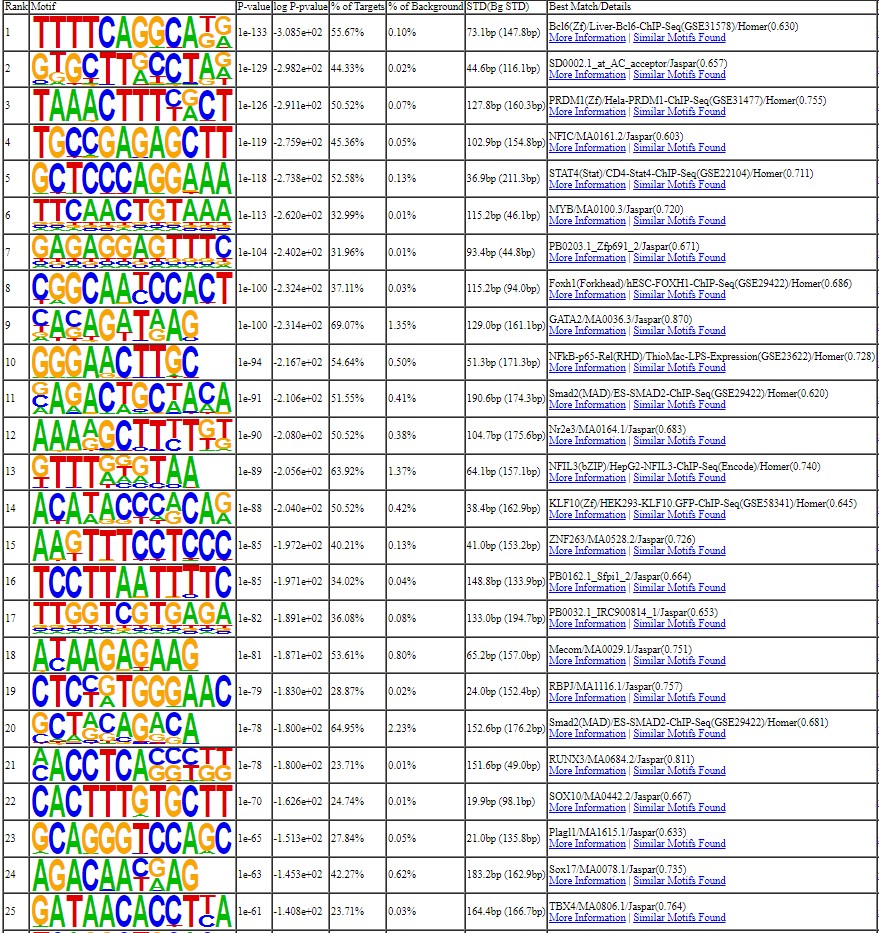


**Figure S13** Homer *de novo* motif results for MER61F (default output). Results are ranked by *p*-value.


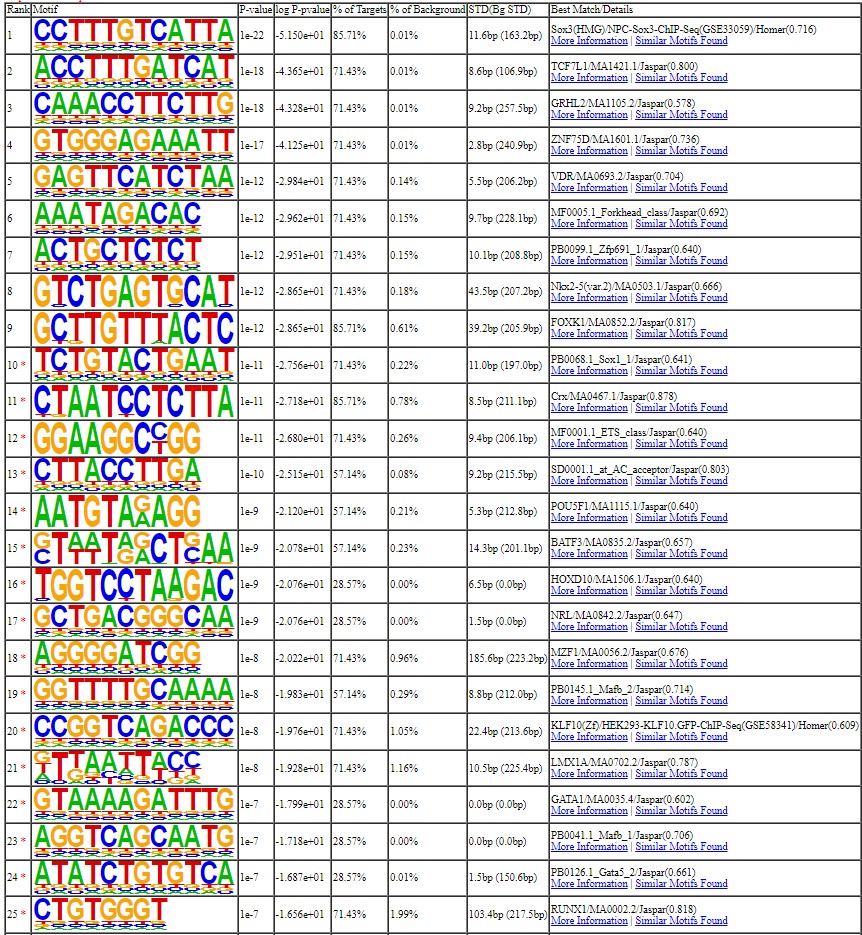


**Figure S14** Homer *de novo* motif results for LTR22C (default output). Results are ranked by *p*-value. Asterisks in “Rank” field indicate potential false positives.


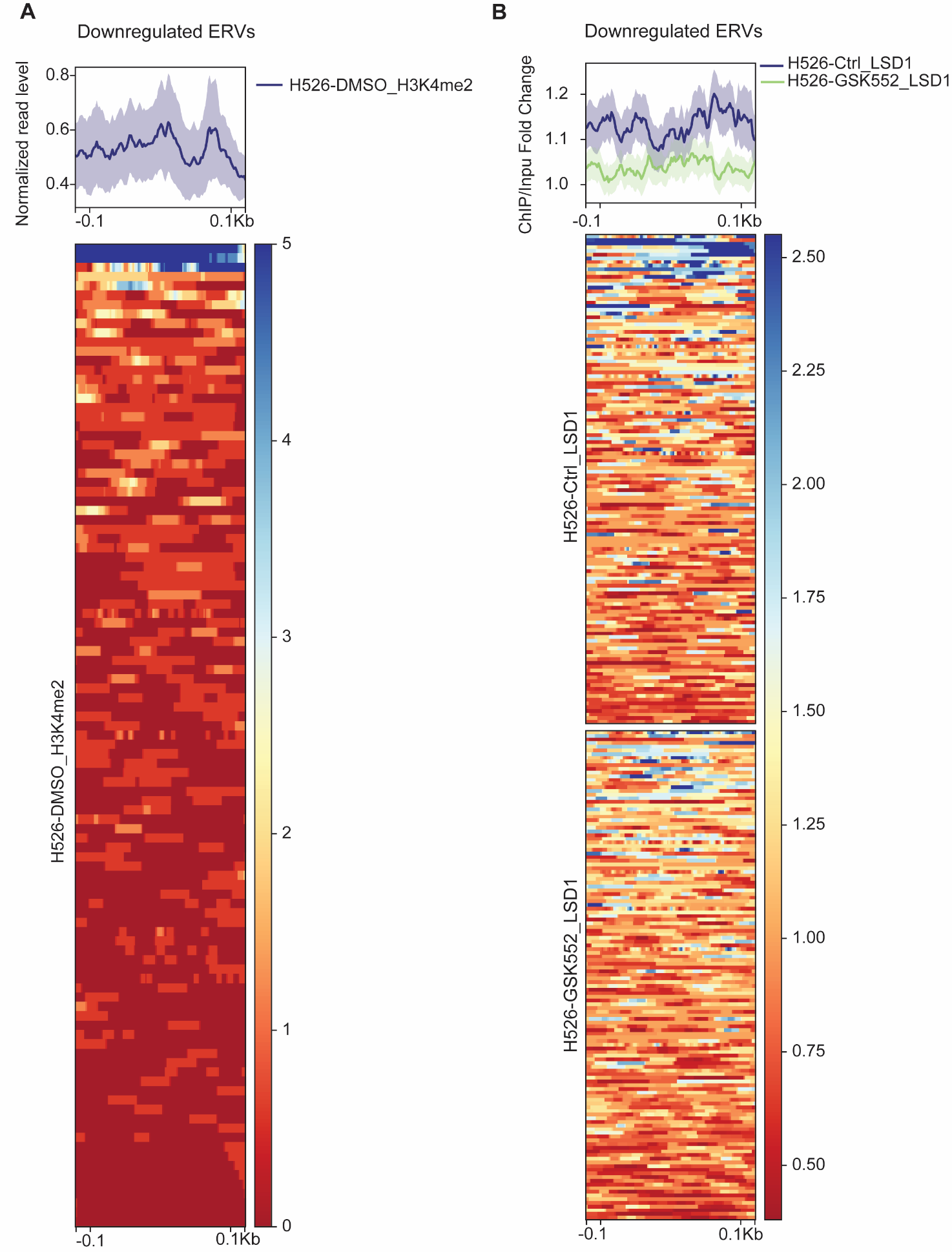


**Figure S15. A)** Mean normalized read levels for H3K4me2 over downregulated expressed ERVs at locus level, in H526 SCLC cell line **B)** Mean genomic signal of ChIP/input ratio over downregulated expressed ERVs at locus level for LSD1 in H526 SCLC cell line in untreated (blue) and treated with LSD1 inhibitor (green) conditions.


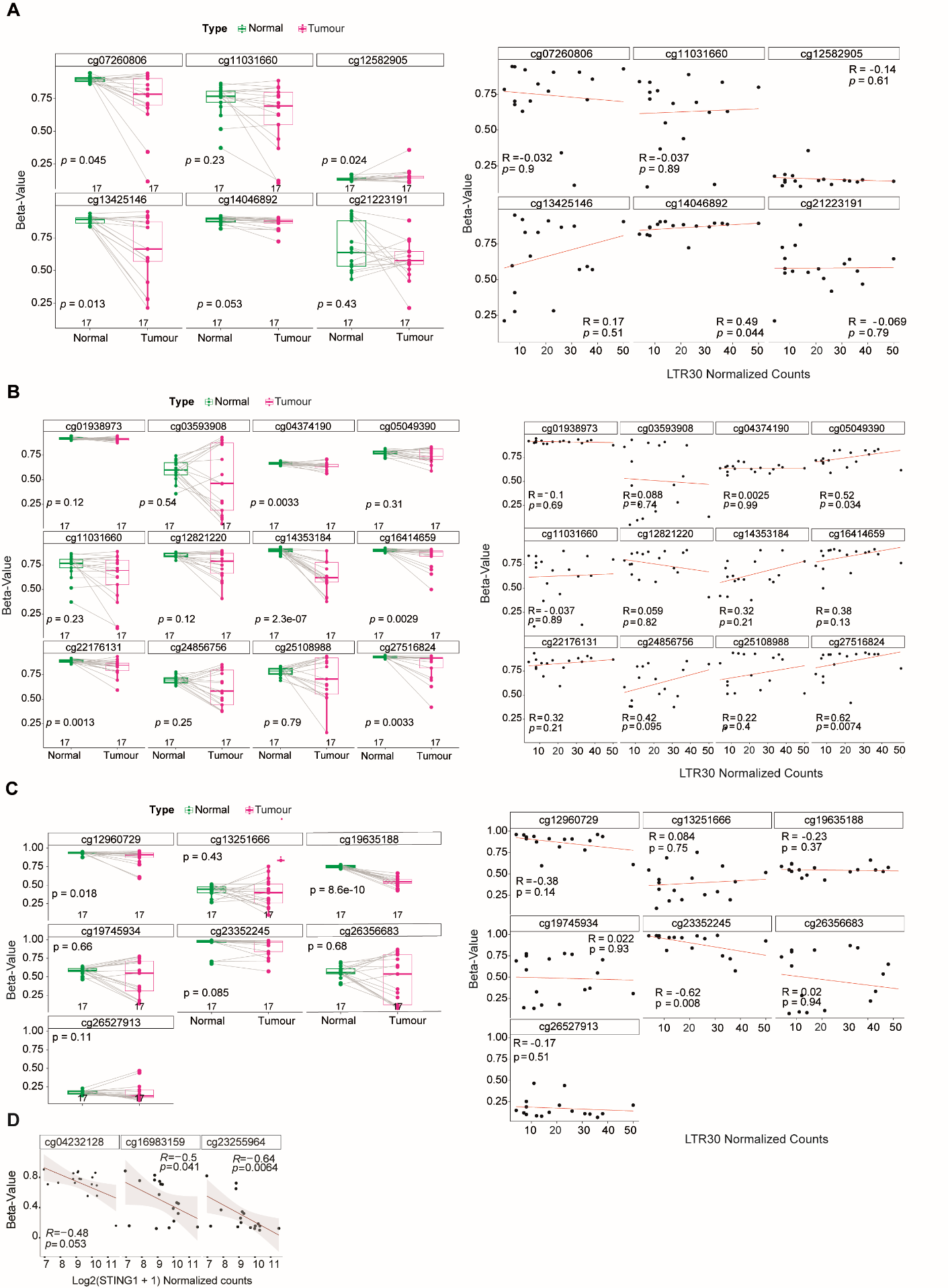


**Figure S16. A-C)** Methylation level (left panel) in SCLC tumours (blue) compared to matched normal samples (red) in cg located in 1000bp upstream **(A)**, genebody **(B)** and 1000bp downstream **(C)** of LTR30 loci. For each cg, correlation between methylation level and LTR30 expression is reported on right panel. **D)** Correlation between methylation levels (y-axis) and STING expression (x-axis) in SCLC tumours for cg located upstream (cg16983159 and cg23255964) and in the gene body (cg04232128) of STING gene.


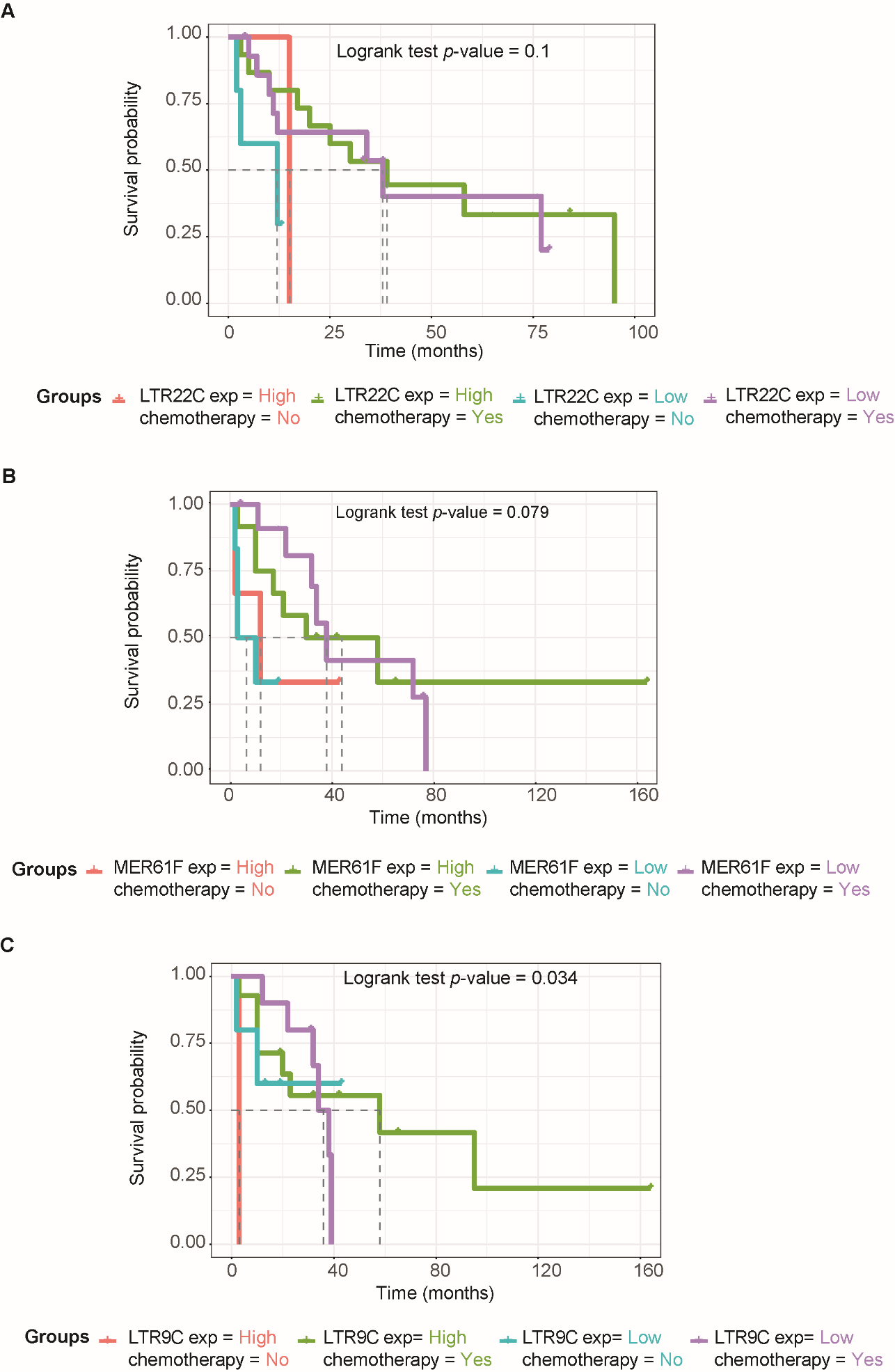


**Figure S17 A-C)** Kaplan-Meier plot of survival estimates of SCLC patients' subgroups (total *n* = 30) depending on expression levels of LTR22C (**A**) MER61F (**B**) LTR9C (**C**) and chemotherapeutic treatments. Log rank test *p*-values are reported. Curves colours are as in legend.
